# Probing the electrochemical behaviour of lithium imide as an electrolyte for solid-state batteries†

**DOI:** 10.1039/d5eb00058k

**Published:** 2025-04-02

**Authors:** Jeremy P. Lowen, Teresa Insinna, Tharigopala V. Beatriceveena, Mark P. Stockham, Bo Dong, Sarah J. Day, Clare P. Grey, Emma Kendrick, Peter R. Slater, Paul A. Anderson, Joshua W. Makepeace

**Affiliations:** a School of Chemistry, University of Birmingham Edgbaston B15 2TT UK j.w.makepeace@bham.ac.uk; b Yusuf Hamied Department of Chemistry, University of Cambridge Cambridge CB2 1EW UK; c I11 Beamline, Diamond Light Source Didcot OX11 0QX UK; d School of Materials and Metallurgy, University of Birmingham B15 2TT UK

## Abstract

All-solid-state batteries utilising a Li-metal anode have long promised to be the next-generation of high-performance energy storage device, with a step-change in energy density, cycling stability and cell safety touted as potential advantages compared to conventional Li-ion battery cells. A key to enabling this technology is the development of solid-state electrolytes with the elusive combination of high ionic conductivity, wide electrochemical stability and the ability to form a conductive and stable interface with Li metal. Presently, oxide and sulfide-based materials, particularly garnet and argyrodite-type structures, have proved most promising for this application. However, these still suffer from a number of challenges, including resistive lithium metal interfaces, poor lithium dendrite suppression (at high current density) and low voltage stability. Here we report the first application of lithium imide, an antifluorite-structured material, as a solid electrolyte in a Li-metal battery. Low-temperature synthesis of lithium imide produces promising Li-ion conductivity, reaching >1 mS cm^−1^ at 30 °C using a modest post-synthetic mechanochemical treatment, as well as displaying at least 5 V stability *vs.* Li^+^/Li. *In situ* electrochemical operation of lithium imide with Li-metal electrodes reveals an apparent 1000-fold increase in its measured conductivity, whilst appearing to remain an electronic insulator. It is postulated that stoichiometry variation at the grain boundary may contribute to this conductivity improvement. Furthermore, the material is shown to possess impressive resistance to hard shorting under high current density conditions (70 mA cm^−2^) as well as the ability to operate in Li-metal battery cells. These results not only highlight the promising performance of lithium imide, but also its potential to be the basis for a new family of antifluorite based solid electrolytes.

## Abstract 2

Lithium imide is an underexplored candidate for an all-solid-state battery electrolyte. Here it is shown to possess many desirable properties and unusual electrochemical behaviour, alongside its first operation in two lithium metal batteries.

## Graphical abstract

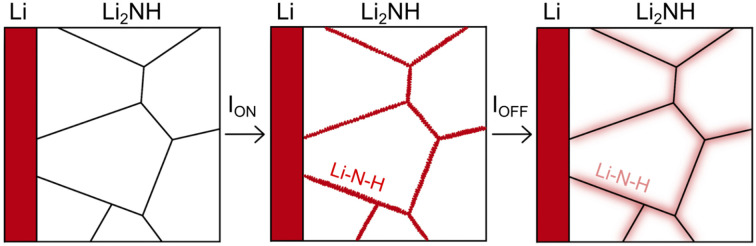

**Broader context**

Solid electrolyte materials offer a promising solution to the safety and performance challenges posed by conventional liquid electrolyte batteries, particularly the issue of metal dendrite formation. However, achieving all of the demanding requirements for solid electrolytes—high ionic conductivity, low electronic conductivity, good processability, and excellent stability under operating conditions—remains a significant challenge. In this study, we introduce lithium imide, a relatively unexplored ionic conductor with promising properties for solid-state battery applications. The material demonstrates excellent compatibility with lithium metal, high ionic conductivity, and a low-temperature synthesis route compared to other leading electrolytes. Interestingly, the material's conductivity increases dramatically upon cycling with lithium, reaching liquid-like levels. This unexpected behaviour is hypothesized to result from compositional changes at the grain boundaries, forming more disordered structures. Given previous exploration of lithium imide materials in the context of hydrogen storage, this study lays the groundwork for a novel family of solid electrolytes based on lithium imide.

The growing demand for batteries with high power and greater energy density is driving the push for adopting lithium metal anodes in the next generation of Li-ion battery technology.^1,2^ This transition is however hindered by a number of challenges including lithium dendrite propagation, the formation of ‘dead’ lithium, and chemical incompatibility with current liquid electrolytes, which leads to uncontrolled solid electrolyte interphase (SEI) growth during cycling.^1–3^ A key strategy towards resolving these issues is to replace organic liquid electrolytes with a solid ceramic or polymer alternative forming a so-called all solid-state battery (ASSB).^4–6^ Employing a solid-state electrolyte (SSE) could offer chemical compatibility with lithium metal, provide a mechanical barrier to dendrite growth, enable the use of future high-voltage cathodes, and address the safety concerns associated with current liquid electrolytes.^4,7^ Finding a suitable SSE material is not, however, straightforward; the performance of the electrolyte must meet a number of strict requirements including:

• High ionic conductivity of at least 0.1 mS cm^−1^ at room temperature.^7,8^

• Electronically insulating.^7,8^

• A wide electrochemical stability window comparable to or greater than that of a conventional liquid electrolyte (up to ∼4.2 V *vs.* Li^+^/Li) with a reduction potential close to that of lithium metal (0 V *vs.* Li^+^/Li).^7–9^

• Low resistance interfaces with both lithium metal and the relevant cathode.

• Lightweight, cheap, and easily accessible through commercially viable synthesis routes.

To date, numerous ceramic material types (ranging from oxides to sulfides) with a variety of structures (*e.g.* Garnet, LISICON, Argyrodite) have been researched for this application, though the majority have yet to reach the market.^10–15^ Even the most extensively researched materials, where impressively high ionic conductivities have been recorded, still have significant challenges to their practical application. Oxide-based materials, such as lithium garnets, are often mechanically hard and form surface lithium carbonates, consequently exhibiting poor wettability with lithium metal and highly resistive interfaces.^16,17^ Sulfide-based materials, whilst softer, display a low upper-voltage stability and are extremely moisture sensitive.^18^ Fluorite-structured oxides and fluorides have long been researched in the context of solid oxide fuel cells and solid-state fluoride batteries, due to their high ambient temperature anionic conductivity.^19–23^ Despite this, comparatively little attention has been paid to corresponding lithium antifluorite materials for Li-ion solid electrolyte application, aside from lithium nitride chloride systems and recent phosphorus-doped lithium sulphide.^24–26^

Lithium imide (Li_2_NH), a nitrogen-based complex metal hydride with an antifluorite-type structure, has previously been explored for its readily-reversible hydrogen storage reaction and impressive catalytic activity for ammonia decomposition.^27–30^ A key characteristic driving the performance of this material in these applications is its high reported lithium-ion conductivity (10^−5^–10^−4^ S cm^−1^), yet only one study has investigated the electrochemical characteristics of the material.^31–34^ This may relate to initial suggestions of a narrow operational voltage range, though these were not experimentally verified. Indeed, subsequent studies of lithium imide point towards a promising set of properties: it is thermally stable up to 600 °C, has an apparently wide electrochemical stability window and ^7^Li NMR data suggest the main charge carrier to be Li^+^, signifying that Li_2_NH is likely electronically insulating.^35,36^ Molecular dynamics simulations have indicated that Li^+^ diffuses *via* an interstitialcy-type mechanism, intrinsically linked with the rotation of N–H bonds within the structure.^37,38^ Although moisture sensitive, Li_2_NH can reportedly be formed in as little as 10 minutes at 210 °C and is composed of largely abundant and lightweight elements, making it more suitable for low cost and high energy density systems as compared to other archetypal SSEs.^39^ Herein we demonstrate the significant potential of Li_2_NH as a SSE, with a new highest recorded bulk ionic conductivity for the material (>1 mS cm^−1^ at RT), wide electrochemical stability window (≥5 V) and excellent high current density operational capability (at up to 70 mA cm^−2^). We demonstrate that *in situ* operation of a Li_2_NH SSE using a Li-metal symmetric cell induces a metastable further increase in the conductivity (>10 mS cm^−1^) without bulk structural changes to the material, indicating possible activation of fast grain boundary diffusion. Operation of hybrid solid-state batteries utilising a Li_2_NH SSE and Li-metal anode is also achieved with two separate cathode materials (LiFePO_4_ and TiS_2_). This material therefore represents a promising new system for achieving high-performance solid-state Li-metal batteries.

## Structure and synthesis

**Fig. 1:**
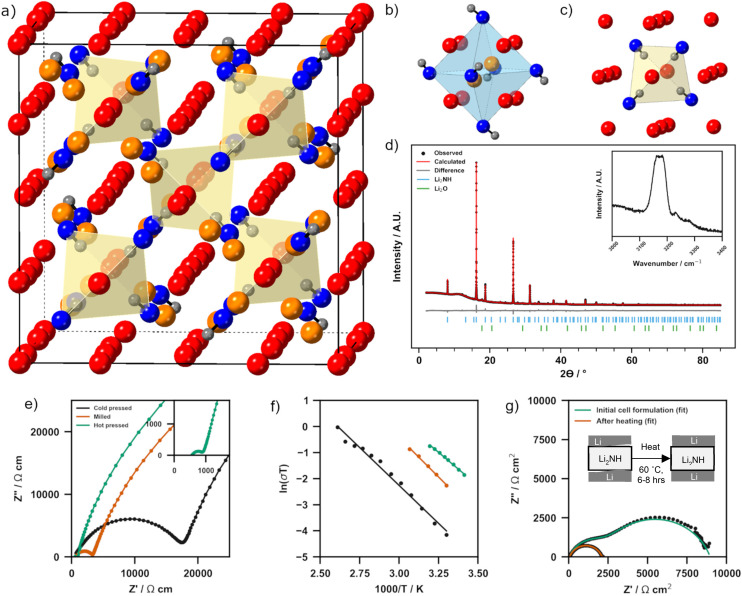
(a) *Fd*3̄*m* structure of stoichiometric Li_2_NH with tetrahedral Li ions shown in red, interstitial Li ions in orange, N in blue and H in grey. (b) Octahedral hole occupied by interstitial Li-ions (c) tetrahedral coordination of local N–H bonds towards vacancy left by Li displacement. (d) Powder XRD pattern including experimental data and Rietveld fit of Li_2_NH with inset Raman spectra. (e) Nyquist impedance spectra of Au|Li_2_NH|Au cells at 30 °C with different pellet preparation strategies. (f) Arrhenius plots of each Au|Li_2_NH|Au cell. (g) Nyquist impedance spectra of a Li|Li_2_NH|Li cell before and after simple heat treatment. Cold-pressed imide was used. A schematic of proposed interface stabilisation is shown.

Lithium imide is widely accepted to adopt a cubic antifluorite-based structure, although there are differing reports as to whether at room temperature this is a simple antifluorite cell with disordered N–H orientations (*Fm*3̄*m* symmetry) or a larger superstructure (*e.g. Fd*3̄*m* symmetry, Fig. 1a) with ordered displacement of Li-ions into octahedral holes (Fig. 1b) and tetrahedral coordination of the lithium vacancy by N–H groups (Fig. 1c).^40–43^ In essence, this structure represents an ordered Frenkel defect variation on the classic antifluorite structure. Given the propensity for Li_2_NH to form antifluorite-structured *Fm*3̄*m* symmetry solid solutions with other N–H based materials, it is unlikely that there are two room temperature polymorphs of Li_2_NH. Instead, experimental reports of the disordered structure for stoichiometric Li_2_NH are much more likely off-stoichiometry due to amide (NH_2_^−^), nitride (N^3−^) or hydride (H^−^) impurities associated with the synthesis method.^31,44–46^ Multiple molecular dynamics and density functional theory simulations investigating the structure of Li_2_NH support this assertion, indicating that stoichiometric Li_2_NH should take the ordered superstructure at room temperature.^37,47–50^ It should be noted that previous reports of the Li-ion conductivity of Li_2_NH have almost exclusively been from samples with this disordered structure and not therefore stoichiometric Li_2_NH. The synthesis of Li_2_NH was achieved using the solid-state reaction of Li_3_N with LiNH_2_ first reported by Hu and Ruckenstein.^39^ The powder X-ray diffraction pattern of Li_2_NH is displayed in Fig. 1d. The peak at 6.9° corresponds to the (111) reflection characteristic of the 2*a* × 2*a* × 2*a* antifluorite superstructure of stoichiometric Li_2_NH. Rietveld analysis was performed using a previously reported *Fd*3̄*m* structure to fit the Li_2_NH phase.^43^

The inset Raman spectrum displays a broad peak at approximately 3180 cm^−1^ corresponding to the linear imide stretch and is consistent with previously reported spectra.^34,45,51^ Two minor peaks at 3240 cm^−1^ and 3275 cm^−1^ are likely indicative of very minor levels of residual amide ions left in the solid solution.^45^ Compared to other solid electrolyte preparations, the synthesis conditions for Li_2_NH are very mild. For example, typical garnet-type oxides often require multiple firings at temperatures >900 °C to complete their synthesis, whilst in this synthesis the sample is calcined in a single step at only 325 °C.^52^

## Ionic and electronic conductivity

The ionic conductivity of as-prepared, cold pressed Li_2_NH was determined at 30 °C *via* electrochemical impedance spectroscopy (EIS) on an Au|Li_2_NH|Au cell. The Nyquist plot for this measurement is displayed in Fig. 1e and the full Nyquist plot with equivalent circuit model is found in ESI Fig. 1† (results of fitting found in ESI Table 1†). One semi-circle may be observed which is assigned to the impedance associated with the combined bulk/grain boundary transport, followed by a spike observed at high frequency, which is characteristic of Li_2_NH–Au interface charging. This blocking behaviour is a good indication that Li_2_NH is a purely ionic conductor. The conductivity was calculated using the total bulk/grain boundary contribution and was found to be 0.054 mS cm^−1^. This is of the same order as the previous literature for this phase.^34^ A potential detriment to the total conductivity of this system is likely the porosity of the cold-pressed pellet which had a relative density of 76.5%. The electronic conductivity and transference numbers of Li_2_NH were determined using DC polarisation on the same Au|Li_2_NH|Au held at 40 °C (ESI Fig. 2†). The electronic conductivity was calculated to be 1.2 nS cm^−1^ whilst *t*_i_ was found to be 0.998, confirming that all charge transfer may be attributed to ionic movement and that Li_2_NH is an electronic insulator.

Given that the ionic conductivity for cold-pressed Li_2_NH does not meet the threshold for SSE application we have pursued further pellet preparation strategies to improve this property. Fig. 1e displays Nyquist plots of these other preparations at 30 °C. Full Nyquist plots with equivalent circuit fitting can also be found in ESI Fig. 1† (fitting results in ESI Table 1†). Similar to cold-pressed Li_2_NH, one semi-circle and spike can be observed for each preparation. A pre-treatment of Li_2_NH powder through low-energy ball milling before cold pressing was found to improve the conductivity to 0.34 mS cm^−1^, whilst retaining a similar pellet relative density of 77%. This improvement in conductivity is likely due to improved grain boundary diffusion, potentially through inducing disorder from milling. Similar effects from milling have been reported for fluorite-based materials, however, to our knowledge this is the first report of this behaviour for an antifluorite material.^53^ Hot pressing Li_2_NH at 325 °C for just one hour has an even greater effect than milling, improving the conductivity to 1 mS cm^−1^ (Fig. 1e inset), the highest recorded conductivity for this material at room temperature. These improvements are likely due to morphological effects as well as a gain in pellet relative density to 85% during hot pressing. Arrhenius plots for the conductivity of all three pellet preparations across a range of temperatures are shown in Fig. 1f. The activation energy for lithium diffusion was calculated to be 0.50(2) eV for cold pressed Li_2_NH, 0.52(2) eV for milled + cold pressed and 0.44(1) eV for hot pressed Li_2_NH. These values are of the order of previously reported values for Li_2_NH.^32–34^ The lower activation energy for hot pressed Li_2_NH is likely a reflection of improved grain boundary diffusion and the lower porosity of the pellet. Given the conductivity gains with these relatively simple physical treatments, it is likely that further optimisation of the sample morphology will yield additional improvements.

## Li-metal interface

Fig. 1g displays Nyquist plots for a Li|Li_2_NH|Li cell at 30 °C before and after heat treatment. Upon initial construction of the cell a large interfacial impedance is observed (∼3308 Ω cm^2^) between the Li_2_NH and Li-metal. However, a simple heat treatment of just 60 °C for 6–8 hours largely eliminates this interfacial resistance (∼47 Ω cm^2^). Equivalent circuit fitting results of these spectra may be found in ESI Fig. 3 and ESI Table 2.† The conductivity of the cell post heat-treatment is found to be in good agreement with Au|Li_2_NH|Au data at 0.059 mS cm^−1^. Post thermal treatment X-ray diffraction analysis (ESI Fig. 4†) displays that no crystalline impurities are formed during this procedure, indicating that this process is likely to be either a mechanical wetting of the interface or a surface-confined reaction which improves conductivity. A proposed mechanical interface formation process is displayed as a schematic in Fig. 1g.

## Electrochemical stability

**Fig. 2:**
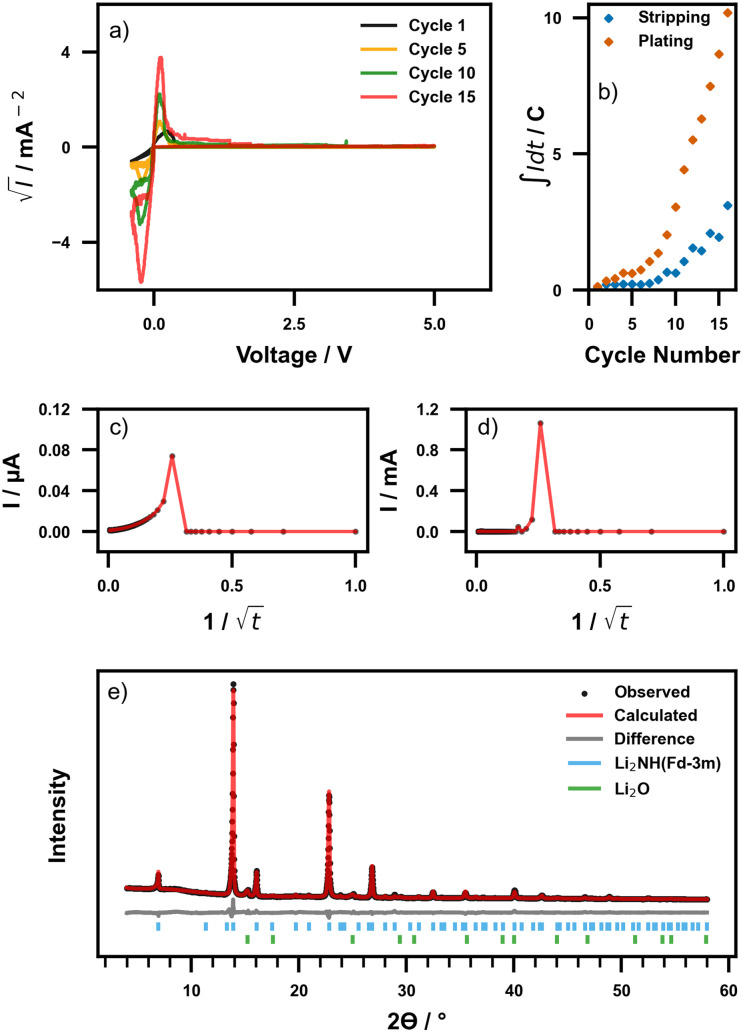
(a) Cyclic voltammograms of a Li|Li_2_NH|Steel cell at 40 °C. (b) Integrated current *vs.* cycle number for stripping and plating. (c) DC polarisation taken (c) before and after (d) cycling. (e) Post-CV (16 completed cycles) powder XRD pattern of material after cell disassembly.

The electrochemical stability of Li_2_NH was assessed *via* cyclic voltammetry (CV) between −0.5 V and 5 V *vs.* Li/Li^+^ on a Li|Li_2_NH|Steel cell where the Li-metal electrode was melded to the Li_2_NH pellet *via* the method used above. No extra pressure was applied to the cell beyond the spacer and spring used in fabrication. The cyclic voltammograms for this measurement are displayed in Fig. 2a. Anodic and cathodic currents corresponding to lithium stripping (positive current, Li → Li^+^ + e^−^) and lithium plating (negative current, Li^+^ + e^−^ → Li) can be observed near 0 V. No currents corresponding to electrolyte decomposition were observed over 16 cycles, indicating that Li_2_NH is electrochemically stable across the measured voltage range, or at least forms a stable electrode–electrolyte interphase. However, it can be observed that the currents corresponding to lithium stripping/plating increase with increasing cycle number. This has previously been ascribed to an improving interfacial morphology between the SSE and Li-metal during cycling, however, it is also possible that the ionic conductivity of the cell is improving through another mechanism, allowing more lithium to diffuse each cycle.^54^

The rate of increase in plating/stripping currents with cycle number follows two regimes, as displayed in Fig. 2b. Initially (cycles 1–7), the proportion of lithium stripped/plated increases slowly, followed by a rapid increase (cycles 7–16). In this second regime, the ratio of stripping peak area to plating peak area appears to stabilise (ESI Fig. 5†). By analysing the difference in the total amount of lithium stripped and plated it was found that 5.2% more lithium is plated than stripped (calculated as a percentage of the number of moles of lithium in Li_2_NH). This discrepancy may be explained either by the formation of dead lithium as a result of loss of contact at the Li–Li_2_NH interface or may be representative of a chemical change in the cell. A loss of lithium could for instance indicate the formation of an imide-amide phase (Li_2−*x*_NH_1+*x*_) which are well known and exist over a wide stoichiometry range where up to two-thirds of imide ions can be replaced by NH_2_^−^ within the Li_2_NH structure.^31^

It is clear that there is a mechanical and/or chemical interaction between lithium metal and Li_2_NH. To probe this interaction a DC polarisation experiment using a voltage of 0.5 V was undertaken both before and immediately (within 2 minutes) after the CV experiment, with data displayed in Fig. 2c and d. The plateau currents imply an electronic conductivity pre-CV and post-CV of 0.45 nS cm^−1^ and 54 nS cm^−1^, respectively (see ESI Fig. 6†), indicating that the cell is still electronically insulating with a small increase, possibly reflecting lithium penetration reducing the effective thickness of the pellet. This is also reflected in the calculated transference numbers pre- and post-CV, where *t*_i_ is found to be 0.981 and 0.999 respectively, indicating dominant lithium-ion conduction. Post-CV there is a far greater spike in current upon initial polarisation (74 nA pre-CV to 1.06 mA post-CV) and a much faster relaxation period compared to the pre-CV measurement. Fitting of both sets of data using exponential decay functions revealed a decrease in this current relaxation period of 4.48(6) s pre-CV to 2.28(1) s post-CV (ESI Fig. 6 and ESI Table 3†). Given that this spike is typically associated with the initial movement of ionic charge carriers upon polarisation this interaction is therefore indicated to result in an overall increase in the ionic conductivity of the cell. Cell disassembly and subsequent X-ray diffraction measurement (Fig. 2e) again indicates no change in the bulk structure of Li_2_NH, nor formation of impurities detected during this process. Given the lack of apparent bulk chemical change, several possible mechanisms for this increased conductivity are conceivable:

1. An electrochemically-driven chemical or morphological process resulting in a more conductive electrode–electrolyte interface.

2. An *in situ* reversible order–disorder phase transition of Li_2_NH to a simpler antifluorite cell (*Fd*3̄*m* → *Fm*3̄*m*) resulting in increased ionic diffusion as theorised in computational studies.^50^

3. Grain boundary/particle surface stoichiometry variation on a local scale undetectable *via* a bulk technique such as diffraction.

It is possible that the discrepancy in lithium stripped and plated reflects a change in the lithium content at the surface or grain boundaries of Li_2_NH particles. Fluorite-type anionic conductors have been found to have increased ionic mobility through the grain boundaries due to stoichiometry variation.^53,55,56^ Furthermore, recent *ab initio* simulations of a Li_2_NH surface catalysing ammonia decomposition with subsequent formation of a non-stoichiometric imide-amide particle surface results in a highly disordered, quasi-liquid surface, where fast-ionic diffusion is extremely plausible.^57^ While these simulations were at 500 °C, it is conceivable that similar particle surface stoichiometry variation under electrochemical conditions might induce an analogous effect, resulting in ‘ionic highways’ along the grain boundaries of Li_2_NH particles.

## Lithium stripping and plating

**Fig. 3:**
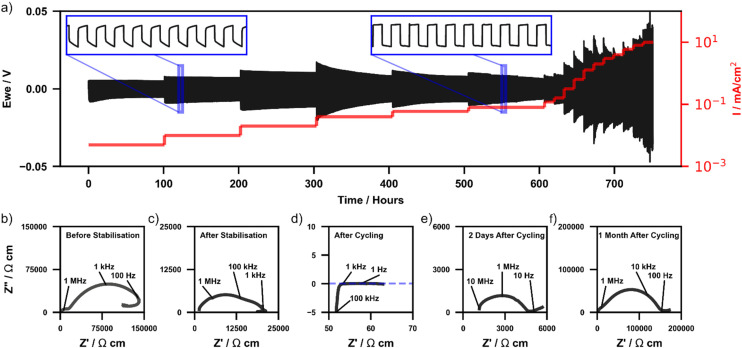
(a) Lithium stripping and plating data of a Li|Li_2_NH|Li cell cycled at 40 °C at various increasing current densities. The positive and negative currents are not displayed to not obscure the voltage data, a line indicating the set current density is instead used alternating between positive and negative currents in 15-minute intervals. Nyquist plots of cell (b) before interface stabilisation, (c) after interface stabilisation, (d) immediately post-cycling, (e) 2 days after cycling, and (f) 1 month after cycling.

To assess the long-term stability of Li_2_NH in contact with a lithium metal anode under battery operating conditions and further understand the interaction between the two materials, lithium stripping and plating experiments (S&P) under constant current conditions were conducted on a Li|Li_2_NH|Li cell. Fig. 3a displays the time-dependent voltage profile of the cell at 40 °C cycled for 200 cycles at 5, 10, 20, 40, 60 and 80 μA cm^−2^ and then for 25 cycles at increasing current densities up to 10 mA cm^−2^. An inset image displays a zoomed-in view of several voltage profiles at a current density of 10 μA cm^−2^ and 80 μA cm^−2^. Under these conditions Li_2_NH is shown to undergo over 750 hours of cycling (>1250 cycles) without a dramatic drop in current that might indicate a hard short-circuit. However, during 40 μA cm^−2^ cycling, a slow decrease in the voltage profile is observed resulting in a lower stable cycling voltage. This voltage drop corresponds to the overall cell resistance reducing each cycle and is also observed at higher current densities at accelerated rates. Whilst this could be the result of slow dendrite penetration it is also consistent with observations from the CV experiments (Fig. 2) that ionic conductivity increases whilst cycling with a Li-metal electrode.

EIS measurements (Fig. 3b–f) were completed both before and after S&P to understand further the effect on conductivity whilst cycling with Li-metal (Bode plots and phase angle data of each EIS measurement are included in ESI Fig. 7†). Fig. 3b and c show the Li|Li_2_NH|Li cell Nyquist plots before interface stabilisation and post-thermal treatment respectively. Before stabilisation two diffusion mechanisms are observed, one at 10^3^–10^4^ Hz ascribed to interfacial diffusion with Li-metal, and another at ∼10^7^ Hz (with a phase angle of −45°) assigned to bulk diffusion. After stabilisation the interfacial component disappears. In both cases a low frequency inductive loop is observed. The exact cause of this remains ambiguous however these have been ascribed in other systems to interfacial stoichiometry variation and may reflect a similar effect at the surface of the Li_2_NH particles.^58,59^Fig. 3d displays the Nyquist plot of the cell immediately after cycling where a decrease in cell impedance by a factor of ∼1000 corresponding to a conductivity of >10 mS cm^−2^ is observed. The high frequency bulk diffusion process observed before cycling is no longer present, replaced by a process with a phase angle of 90°. Minimal capacitive behaviour is seen (ESI Fig. 8†), indicating that charge is able to move freely through the cell.^60^ Combining these two observations points towards the change observed being surface related. The conductivity calculated post-cycling is on the order of the best solid-state ionic conductors known to date, however, the exact nature of this improvement is enigmatic. The other possibility is the formation of soft short circuits resulting in mixed ionic-electronic conductivity, which could result in a similar impedance spectrum to that shown in Fig. 3d, though the post-CV DC polarisation experiments indicated minimal evidence for this.

Fig. 3e and f display EIS measurements on the same cell after set periods of time at rest. Over this time the cell is observed to display relaxation/recovery behaviour. This is reflected in both an observed recovery of cell resistance as well as in the phase angle data, where two days after cycling the high-frequency angle returns to around 45° indicating a bulk diffusion limited process. After one month, the low frequency diffusion process assigned to a Li_2_NH|Li interfacial component returns, whereby the impedance spectrum is comparable to that of prior to stabilisation. This implies a dynamic and metastable interfacial process underpins the increased conductivity observed. Repeat experiments performed on separate cells show similar conductivity improvement and corresponding recovery behaviour (ESI Fig. 9†). Synchrotron X-ray diffraction of the post-cycled material shows again no change to the bulk structure nor appreciable formation of impurities (ESI Fig. 10†).

Given the limitations of the structural changes which can be ascertained from average structure measurements, solid state NMR spectroscopy was employed as a means of assessing local changes in the material. ESI Fig. 11† displays ^7^Li and ^1^H spectra for pristine and post-cycled Li_2_NH. The ^7^Li NMR spectrum of the pristine Li_2_NH consists of a sharp Lorentzian line centred at 3.30 ppm with an underlying broader component centred at 3.40 ppm, suggesting two separate Li ion environments, one more mobile than the other. The post-cycled sample displays a shift in the broader component to 3.03 ppm, indicative of a minor change in this environment post cycling and potentially minor stoichiometry variation. It should be noted that changes in signal intensity between the two materials remain inconclusive due to measurements having been run at different magnetic fields (the cycled sample having therefore experienced greater polarisation) and on different probes (with different *Q*-factors). However, qualitatively we observe a drop in the ^7^Li signal intensity between the pristine and cycled material, perhaps suggesting Li loss. A change is also observed in the ^1^H NMR spectra where the pristine sample shows a variety of environments associated with the NH_*x*_ groups: one at −4.35 ppm which is likely the imide groups, one at −1.53 ppm which may be residual amide groups as discussed above, and some very low intensity resonances at 3.97 ppm, which are in the chemical shift region of saline hydrides.^61^ Post cycling, the main peak shifts to − 5.13 ppm and the second peak to −2.28 ppm again indicating stoichiometry variation compared to the pristine sample (discussed in further detail below). Furthermore, the echo delay in the Hahn echo pulse sequence can be increased to filter out fast relaxing components (*i.e.* having a short transverse, *T*_2_, relaxation time). Such *T*_2_-filtered ^1^H spectra (ESI Fig. 12†) highlight a higher proportion of less mobile hydrogen environments in the post-cycled sample. Both amide and hydride anions are expected to be less mobile than the free rotation of the imide group within Li_2_NH, suggesting the presence of one/both of these anions in low quantities. Whilst these data indicate that the observed process is potentially non-stoichiometric in its nature, the metastability demonstrated by the observed recovery behaviour limits the insight offered by *ex situ* analysis on the mechanism of the conductivity increase. Therefore, *in situ* diffraction and NMR measurements were performed to gain temporal resolution.

## *In situ* X-ray diffraction and solid-state ^7^Li NMR

**Fig. 4:**
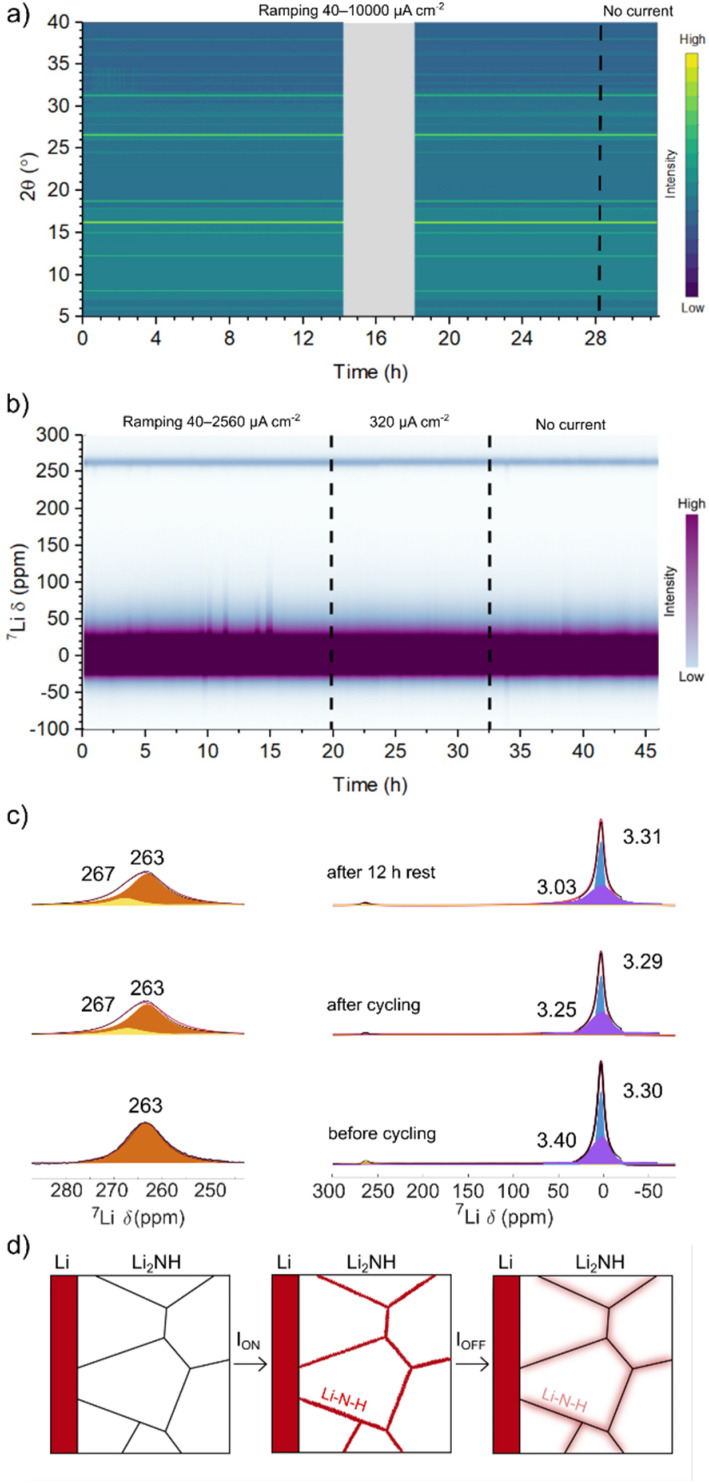
Contour plots of (a) synchrotron X-ray diffraction data and (b) ^7^Li SS non-spinning NMR spectra from Li|Li_2_NH|Li coin cells during lithium stripping and plating at the indicated current densities, with (c) key SSNMR spectra fitted with a CSA static model. A schematic of the proposed mechanism of enhanced grain boundary Li-ion conduction through non-stoichiometric Li–N–H phase formation at grain boundaries, and subsequent diffusion into the grains is shown in (d).

An accelerated S&P experiment with *in situ* synchrotron diffraction was carried out to assess bulk structure behaviour during cycling, particularly with regard to potential metastable phases or stoichiometry variation. In this case cycling was started at 40 μA cm^−2^ and doubled every 3 hours up to 10 mA cm^−2^. The electrochemical data for this experiment are displayed in ESI Fig. 13† with a contour plot of the observed diffraction data at each indicated current density displayed in Fig. 4a (example diffraction pattern shown in ESI Fig. 14†). Again, despite the accelerated timescale of this experiment, there is no sudden and sustained drop in voltage to indicate a short circuit and a similar lowering of resistance is observed. The diffraction data display no change in the average structure of Li_2_NH over the course of this experiment. Peaks corresponding to a small Li_3_N component are present throughout and are thought to originate on the outside lithium metal surface closest to the Kapton window of the cell, where visible nitriding could be seen. ESI Fig. 15† displays a separate cell run on a further accelerated timescale (current density doubled every hour) along with cycling for 18 hours at 10 mA cm^−2^. The lowering of resistance and stable cycling behaviour at 10 mA cm^−2^ is well demonstrated here together with no corresponding change in average structure. After a rest period (approximately 6 hours) this cell was rerun on a similar programme from 40 μA cm^−2^ up to 70 mA cm^−2^. At the highest current densities, a large polarisation in the voltage profile of each cycle is observed along with variation in the measured diffraction signal for Li metal, but it again appears that there is no hard short circuit. The flattened voltage profiles for the second cell which evolve at 10 mA cm^−2^ (Fig. S15†) have been suggested to reflect the presence of soft-short circuits,^62^ although it is difficult to determine this unambiguously. The lack of structural variation whilst cycling excludes the possibility of a bulk phase transition of Li_2_NH from *Fd*3̄*m* → *Fm*3̄*m*. This, combined with the *ex situ* NMR data, strengthens the hypothesis that the observed increase in conductivity may be due to small-scale stoichiometry variation confined to the grain boundary.

An *in situ*^7^Li NMR S&P experiment was performed to further probe this hypothesis. The electrochemical data for this experiment are displayed in ESI Fig. 16† with a contour plot of the observed NMR spectra in Fig. 4b. The spectra show a strong signal centred close to 0 ppm from Li_2_NH and a smaller signal from Li metal at around 260 ppm. In this case, the cell was cycled at each current density for 3 hours then doubled, starting from 40 μA cm^−2^ up to 2.56 mA cm^−2^. The cell was then cycled at 320 μA cm^−2^ for 12 hours before a 12-hour rest period. The electrochemical data can be observed to be noisy, possibly indicating dendrite formation although this is countered by the observed recovery of the voltage profiles. No significant change in the shift of the Li_2_NH peak is observed whilst current is applied, indicating that the local Li environment within Li_2_NH does not change drastically during cycling. The absence of significant shifts outside the diamagnetic region (except for Li metal itself) also confirms that the sample does not become electronically conductive.^63^ The development of a small Li metal component (∼267 ppm) in addition to the original Li metal signal (∼263 ppm) indicates the expected formation of Li microstructures during cycling. The deposited Li is likely to be rough (the Li–Li_2_NH interphase is quite flat) rather than dendritic, as the latter experiences different bulk magnetic susceptibility effects, since it grows perpendicularly to the Li metal in the cell (and to the applied field), resulting in a larger shift (∼10 ppm) from that of the bulk Li signal.^64^

Monitoring the variation in the integral of the Li metal and Li_2_NH peaks over time (ESI Fig. 17†) reveals initially a drop in the Li-metal signal in the first hour followed by an increase in the next 14 h, this increase being consistent with some formation of Li microstructures at the Li–Li_2_NH interface. A similar drop is observed in the Li_2_NH signal, which then recovers as the current density is doubled from 40 μA cm^−2^ to 80 μA cm^−2^ (a decrease in the cell potential is also observed at this point). The signal then slowly decays throughout the remaining cycling period suggesting potential Li loss from the Li_2_NH phase. The ^7^Li NMR spectra corresponding to pre-cycling, at the final cycle, and after 12 h resting at zero current (Fig. 4c) were fitted using a chemical shift anisotropy (CSA) static model: two components were fitted, one quasi-axial likely corresponding to Li in the interstitial sites (broader, in purple in the spectra) and one rhombic assigned to the Li in tetrahedral sites (narrow and in blue). The fits show that the broad component progressively shifts during cycling (3.40 → 3.25 ppm), with a slightly more significant shift occurring during the rest period (3.25 → 3.03 ppm), while the narrow component remains approximately constant at ∼3.30 ppm.

While it is difficult to unambiguously determine the nature of the different local environments, the observed changes in chemical shift after cycling suggest that the stoichiometry of Li_2_NH varies upon cycling. This may give insight into the observed changes in conductivity and resistance recovery behaviour discussed above. We hypothesise that under applied current or potential there is a stoichiometry variation at the grain boundary leading to a disordering of the surface of the Li_2_NH particles and a highly conductive state, similar to that described for Li_2_NH ammonia cracking catalysts.^57,65^ There is likely some exchange of this highly conductive state with the bulk (reflected by the change in signal before and immediately after cycling). However, the change is metastable and when the electrochemical bias is removed the compositional gradients lessen through diffusion further into the bulk grain, as represented by the greater shift in signal after rest and resulting in the recovery in resistance observed in the EIS spectra (see Fig. 4d for a schematic depiction of this process). We stress that this variation in stoichiometry is very minor and not significant enough to alter the average structure of the material and hence we do not observe this relaxation *via* diffraction. Additional surface sensitive Dynamic Nuclear Polarisation (DNP) NMR experiments could help confirm the local environment at the grain boundary.

As this potential stoichiometric variation occurs whilst in contact with lithium metal, it is prudent to consider any possible reactions between the two materials. Eqn (1) and (2) detail possible mechanisms for stoichiometric variation.

1 *x*Li + Li_2_NH → Li_2+*x*_NH

2 (*x* + 1)Li_2_NH → Li_2−*x*_NH_1+*x*_ + *x*Li_3_N

Eqn (1) relates to the formation of surface imide-nitride-hydride through reaction with lithium, whilst eqn (2) details the formation of an imide-amide phase and lithium nitride. As such, an assessment of the nature of the stoichiometry variation observed was conducted *via* NMR measurement of two more *ex situ* samples: a lithium imide-amide (Li deficient compared to Li_2_NH, Li_1.917_NH_1.083_) and a lithium imide-nitride-hydride (Li_2.083_NH) (ESI Fig. 18†). Both of these materials also take an antifluorite-type structure and simply represent a shift in bulk stoichiometry compared to Li_2_NH (see ESI Fig. 19† for diffraction and structural data). The ^7^Li and ^1^H NMR spectra of these samples confirm a change in the local structure compared to pristine Li_2_NH (ESI Fig. 11†). For both the imide-amide and imide-nitride-hydride, the ^7^Li spectra contain two resonances (sharp and broad). These are centred at 2.08 ppm (sharp) and 1.56 ppm (broad) for the amide-imide and 3.36 ppm (sharp) and 3.03 ppm (broad) for the imide-nitride-hydride (we note that the linewidth of the imide-nitride-hydride is however ∼2× that of the other samples analysed here). In both samples there is a shift to lower ppm in the broad component as compared to pristine Li_2_NH. Observing the ^7^Li spectra in isolation, the post-cycled material appears to resemble the imide-nitride-hydride most closely, particularly considering the chemical shift of the broad component is identical (3.03 ppm). However, the ^1^H spectra show two resonances centred at −5.59 and −2.74 ppm for the amide-imide and three resonances at −4.98, 0.67 and 3.00 ppm for the imide-nitride-hydride. In this case the ^1^H spectrum of the post-cycled sample most closely resembles the spectrum of the imide-amide: in both spectra the main component (light blue in ESI Fig. 18†) shifted to a more a negative ppm compared to pristine Li_2_NH. Comparison to these fixed off-stoichiometry samples is therefore likely to be a simplification of the overall picture and it is possible that formation of imide-amide and imide-nitride-hydride phases are happening simultaneously during cycling. This is potentially evidenced by the complex range of ^1^H environments revealed in the *T*_2_-filtered experiments. We do however note that observation of Li loss from the Li_2_NH phase in our *in situ* NMR and CV experiments, suggest the change in stoichiometry is on average towards an imide-amide type phase (Li_2−*x*_NH_1+*x*_). Furthermore, it is these lithium imide-amide phases that have been computationally observed to exhibit significant surface disorder.^65^

It is difficult to rule out the possibility that soft-short circuiting might be also contributing to the enhanced conductivity observed in the stripping and plating experiments^66^ and is certainly possible in the higher current density data presented. However, the lack of voltage spikes or sudden drops normally associated with shorting behaviour, the persistent low electronic conductivity measured within a few minutes of the CV experiments, and the absence of dendritic Li microstructures in the *in situ* NMR experiments indicates atypical behaviour for soft-shorting. Furthermore, the system appears quite stable against hard-shorting, which is encouraging. It may be that penetration of Li into the pellet is at the heart of the observed stoichiometry variation along grain boundaries, with rapid reaction of dendrites resulting in more conductive pathways. It is clear that further analysis of this phenomenon is required.

## Li-metal battery proof-of-concept tests

**Fig. 5:**
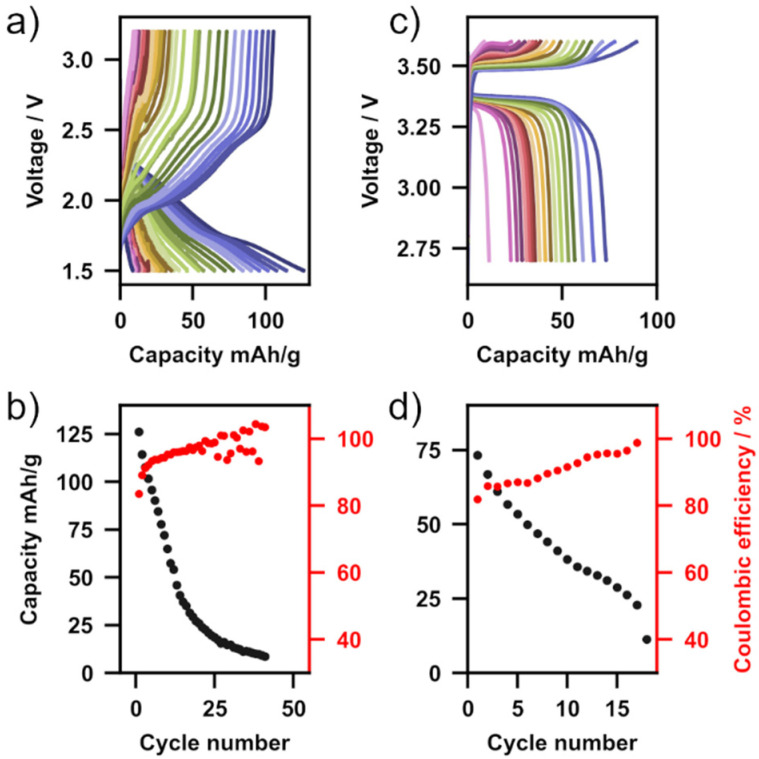
(a) Charge–discharge cycles of a Li|Li_2_NH|TiS_2_ cell at 40 °C, C/48. (b) Capacity and coulombic efficiency *vs.* cycle number of Li|Li_2_NH|TiS_2_ cell. (c) Charge–discharge cycles of a Li|Li_2_NH|LiFePO_4_ cell at 40 °C, C/35. (d) Capacity and coulombic efficiency *vs.* cycle number of Li|Li_2_NH|LiFePO_4_ cell.

The proof-of-concept performance of Li_2_NH in Li-metal battery cells was evaluated using a Li-metal anode and two different cathode materials (TiS_2_ and LiFePO_4_). In both cases a small amount of liquid electrolyte (10 μL, LiPF_6_ in EC : DMC for TiS_2_, LiPF_6_ in EC : EMC with 2 wt% vinyl chloride for LFP) was used to wet the solid electrolyte–cathode interface forming so-called hybrid solid-state batteries. Fig. 5 shows charge–discharge measurements operated at 5 mA g^−1^ for each of these cells (Fig. 5a – TiS_2_, Fig. 5b – LFP) as well as capacity and columbic efficiency as a function of cycle number (Fig. 5c – TiS_2_, Fig. 5d – LFP). With both cathode materials multiple charge–discharge cycles are observed, demonstrating the first application of Li_2_NH as a functioning solid electrolyte in a full cell. With a TiS_2_ cathode, over 40 cycles were completed with a relatively high initial discharge capacity of 126 mA h g^−1^. Capacity fade was observed in both cells, with the TiS_2_ cell terminating after 42 cycles and the LFP cell diminishing after 18 cycles. This cell degradation is likely from the formation of an unstable CEI layer at the Li_2_NH–cathode interface due to a reaction with the liquid electrolyte, and is particularly prominent at voltages above 3.5 V, hence the worse performance of the LFP cells. This is supported by the inferior stability of LFP cells when the 2 wt% vinyl chloride additive (included for more favourable CEI formation) is not used in the liquid electrolyte formulation (ESI Fig. 18†). In either case, no indication of reduction in cell impedance analogous to the S&P experiments was observed, however, it is possible that this is due to the low number of cycles in these battery cycling experiments. As this is the first demonstration of this material in operation, we anticipate that further optimisation of cell formulation both in hybrid and all-solid-state configurations will further improve performance.^65^ At the very least, given the known ability of the antifluorite structure to incorporate amide, nitride, hydride and halides, there is significant potential for a new family of high-conductivity materials to be explored.^31,67,68^

## Summary

The results reported here demonstrate the promising performance of antifluorite Li_2_NH for application as a solid electrolyte in solid-state Li-ion batteries. A high initial ionic conductivity of Li_2_NH is further improved through modest post-synthetic milling and hot-pressing regimes resulting in the highest reported conductivity for this material at 1 mS cm^−1^. High current operation at up to 70 mA cm^−2^ as well as wide voltage stability (0–5 V *vs.* Li/Li^+^) is reported. An interaction with Li-metal electrodes is also observed, with an apparent improvement in conductivity to >10 mS cm^−1^ whilst appearing to remain electronically insulating. This interaction is hypothesised to occur along the grain boundary of the material and may be as a result of stoichiometry variation at the Li_2_NH particle surface producing a highly disordered surface. The impressive performance of this material in these initial investigations suggests the potential for further enhancements. Through the understanding of the wealth of possible composition modifications of lithium imide from the hydrogen storage literature, a new family of imide-based ionic conductors are open for further investigation.

## Methods

### Sample handing

All sample handing was performed in an argon filled glovebox (MBraun, Unilab, <0.1 ppm H_2_O, <0.1 ppm O_2_).

### Synthesis

Synthesis of Li_2_NH was achieved by the solid-state reaction of LiNH_2_ (Sigma Aldrich hydrogen storage grade) with Li_3_N (Sigma Aldrich >99.5%) in stoichiometric quantities.Li_3_N + LiNH_2_ → 2Li_2_NH

The reagents were weighed out in order to form 1 g of Li_2_NH (0.3973 g LiNH_2_ and 0.6027 g Li_3_N). Samples were first hand-ground using an agate pestle and mortar for 2 minutes before being transferred into a Si_3_N_4_ milling jar (volume 45 ml) prefilled with 20 g of 5 mm diameter Si_3_N_4_ milling balls. The jar was sealed and transferred from the glovebox to a Fritsch Pulverisette 7 Premium Line planetary micro mill. The mixture was milled at a rate of 150 rpm for 1 hour. Once returned to the glovebox the resultant mixture was transferred into a quartz tube and fitted with a Young's tap T-piece connected by an Ultra-Torr fitting. This reaction vessel was then clamped into a tube furnace (Lenton Furnaces, LTF 12/25/250 fitted with a Eurotherm 3216P1 controller) and gas lines were attached either side of the Young's tap. Argon gas was then allowed to flow through the tap. The furnace was ramped to 325 °C at a rate of 2 °C min^−1^ and held at that temperature for 12 hours. The sample of Li_1.917_NH_1.083_ was synthesised by varying the Li_3_N to LiNH_2_ ratio to give the appropriate stoichiometry.

For the lithium imide-nitride-hydride sample (Li_2.083_NH), lithium nitride hydride was synthesised by the reaction of lithium nitride with lithium hydride (Sigma Aldrich, 98%) according to the following reaction:Li_3_N + LiH → Li_4_NH.

The powder mixture was milled as above, pressed into a pellet and then heated in a microwave reactor (CEM Discover) for five rounds of 1-minute heating at 300 W under argon flow. The synthesised lithium nitride hydride was then mixed with lithium imide, milled as above and heated to 540 °C under flowing argon for 12 hours (2 °C min^−1^ ramp rate):0.083 × Li_4_NH + 1.917 × Li_2_NH → 2Li_2.083_NH.

### X-ray diffraction

Laboratory powder X-ray diffraction (XRD) measurements were carried out using a Stoe Stadi-P instrument (Mo K_α1_ source) with samples sealed in capillaries made from Cole-Parmer polyimide tubing (0.7 mm internal diameter). Rietveld analysis of XRD patterns was performed using TOPAS Academic software.^69^

### Raman spectroscopy

Raman measurements were taken using Renishaw InVia Raman microscope. Samples were loaded in a borosilicate glass capillary (0.7 mm internal diameter) to an approximate height of 5 mm and sealed with vacuum grease. A laser wavelength of 532 nm was used to analyse the samples. Spectra were taken in the region of 3000 cm^−1^ to 3400 cm^−1^ using a laser power of 0.5% and a typical exposure time of 10 seconds.

### Preparation of Li_2_NH for electrochemical measurements

The Li_2_NH powder was pressed into a pellet (diameter 10 mm, thickness 1–2 mm) under an Ar atmosphere. For Au|Li_2_NH|Au cells, a small amount of vacuum grease was applied to the curved side edge of each pellet before both sides were sputtered with gold using an Agar auto sputter coater placed inside an argon filled glove bag. Sputtered pellets were returned to a glovebox and the gold and grease were removed from the sides to leave gold electrodes only on the faces of each pellet. For Li|Li_2_NH|Li cells, lithium foil (Pi-KEM, 0.25 mm thickness) was cut into 10 mm circles (0.0099 g) using a hole punch and any surface oxidation was removed using a spatula. The Li foil was then applied to both sides of the pellet and light pressure was applied by hand. For Li|Li_2_NH|Steel cells the Li metal was only applied to one side of the pellet. In all cases, each cell was constructed into coin cells for all electrochemical measurements. Coin cell assembly took place by placing each pellet with attached electrodes in a CR2032 cell cap (0.9150 g). A stainless-steel spacer (0.5 mm thickness, 0.7422 g) and spring (0.25 mm thickness, 0.1983 g) were placed on top followed by a CR2032 cell cap with an attached O-ring (0.8853 g). The cell was then sealed using a Hohsen Corp coin cell crimper. Stabilisation of the Li–Li_2_NH interface was conducted on already constructed coin cells by placing cells in an oven (Memmert, UN55) at 60–80 °C for 6–8 hours.

### Electrochemical impedance spectroscopy

Variable temperature electrochemical impedance spectroscopy measurements were taken in a Carbolite VST 12/400 tube furnace heated to a maximum of 110 °C, with the impedance measurements (100 mV perturbation) being taken by a Hewlett Packard 4192A LF between frequencies of 5 Hz to 13 MHz. Measurements on Li|Li_2_NH|Li cells were taken using a Solartron 1260 impedance analyser (100 mV perturbation) from 1 mHz to 10 MHz. Impedance data were analysed using Zview software by Scribner. In general, resistance values for Li_2_NH were taken as the high intercept of the first semi-circle. Where data were fitted to equivalent circuit models, resistance of the material was taken as the combined resistance of *R*1 and *R*2 (see ESI Fig. 1 and ESI Table 1†). Area specific resistance was calculated by normalising Li|Li_2_NH|Li data to the surface area of the pellet (0.7854 cm^2^) and dividing the calculated resistance by 2 to account for the two Li electrodes. For Fig. 3d (ESI Fig. 7†) the resistance was taken as the high intercept of the assumed semi-circle (approximately 61 Ω cm).

### DC polarisation

Electronic conductivity of Li_2_NH was evaluated using DC polarisation experiments. Different voltages (0.5 V & 0.75 V) were applied for a period of 5 hours on an Au|Li_2_NH|Au cell held at 40 °C using a Biologic VMP3. The electronic conductivity was calculated using the following equation:3
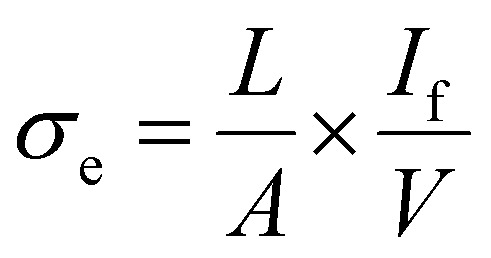
where *L* is the thickness of the pellet, *A* is the surface area of the pellet, *V* is the applied potential, and *I*_f_ refers to the average current of the last half hour of the relaxation period. Transference numbers were calculated from the voltage *vs.* time plots using the following equations:4
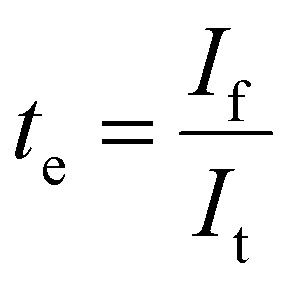

5 *t*_i_ = 1 − *t*_e_

where *t*_e_ and *t*_i_ are the electronic and ionic transference numbers respectively and *I*_t_ is the initial current upon polarisation.

### Cyclic voltammetry

Cyclic voltammetry was conducted on Li|Li_2_NH|Steel cells held at 40 °C using a Biologic VMP3 across a range of voltages from −0.5 V up to 5 V *vs.* Li/Li^+^ at a scan rate of 1 mV s^−1^. Electronic conductivity was evaluated both before and immediately after the CV experiment using the Hebb–Wagner method.^71^ The same cell had a 0.5 V potential applied for 5 hours and the current response measured. The electronic conductivity was calculated as per eqn (3). Transference numbers were calculated as per eqn (4) and (5).

### Lithium stripping and plating

Lithium stripping and plating experiments were conducted on symmetrical Li|Li_2_NH|Li coin cells held at 40 °C using a Biologic VMP3. These symmetric cells were cycled under constant current conditions at various current densities for 15 minutes plus a 10 s rest per half cycle making each full cycle approximately 30 minutes long.

### Synchrotron X-ray powder diffraction

Where appropriate, synchrotron measurements were undertaken on the I11 beamline at Diamond Light Source, with powder samples sealed in 0.5 mm borosilicate capillaries. Lithium stripping and plating experiments were conducted on modified Li|Li_2_NH|Li coin cells with a 3 mm diameter Kapton window to allow for beam penetration. These cells were cycled under identical conditions to the stripping and plating experiments except on an accelerated timescale and using an Ivium Octostat potentiostat. Diffraction data (*λ* = 0.824042 Å) were collected on each of the two cycling cells every 100 s using position sensitive detectors (PSD), with 10 s total collection time per scan. The cells were aligned in the beam relative to the diffraction pattern of a 0.5 mm borosilicate capillary measurement of lithium imide.

### Solid state NMR

Solid-state Nuclear Magnetic Resonance (NMR) experiments were performed on the pristine Li_2_NH powder as synthesized, after cycling (*ex situ*) and *in situ* during electrochemical cycling. *Ex situ* solid state NMR spectra on the pristine material and Li_1.917_NH_1.083_ were acquired on a 11.7 T (*ω*_H_ = 500 MHz) Bruker Avance III spectrometer, while the cycled Li_2_NH spectra were acquired on a 16.4 T (*ω*_H_ = 700 MHz) Bruker Avance III spectrometer. In both cases a Bruker 1.3 mm magic angle spinning (MAS) probe was used with a MAS frequency of 40 kHz. The spectra were externally referenced to glycine (spun at 20 kHz) at 8.00 ppm (*δ*^1^H) and Li_2_CO_3_ at 0.00 ppm (*δ*^7^Li). In all cases, a rotor-synchronised Hahn-echo pulse sequence (90°–*τ*–180°–*τ*–acquire) was used for quantitative measurements. The recycle delay adopted was at least 5 × *T*_1_, the *T*_1_ having been measured *via* a saturation recovery pulse sequence ((sat)_*n*_–*τ*–90°–acquire). For ^7^Li NMR, pulse lengths of 1.0 μs (at 11.7 T) and 2.05 μs (at 16.4 T) and recycle delays of 1.36 s (pristine), 10 s (cycled), 2.65 s (Li_1.917_NH_1.083_) were set; for ^1^H NMR, pulse lengths of 0.87 μs (at 11.7 T) and 2.15 μs (at 16.4 T) and recycle delays of 28.9 s (pristine), 23.4 s (cycled) and 26.5 s (Li_1.917_NH_1.083_) were set. All spectra were scaled according to the mass of the sample and number of residuals recorded.

*In situ* NMR experiments were conducted on a 7.05 T (*ω*_H_ = 300 MHz) Bruker Avance NMR spectrometer equipped with an *in situ* NMR probe (NMR Service GmbH) with automatic tuning and matching capabilities and built-in highly shielded electrochemistry connections. Additional radiofrequency low-pass filters were used on the connection to the potentiostat to prevent interference. A 12 mm inner diameter solenoid coil was used and the cell (made of polyether ether ketone, PEEK) was oriented so that the lithium chips and the electrolyte pellet were parallel to the main magnetic field.^70^ During cycling, NMR spectra were recorded continuously using a one pulse sequence with a pulse length of 5.6 μs and a recycle delay of 1 s, quantitative for Li metal and providing enough signal and time resolution for the solid electrolyte. The time resolution was of ∼1 min per spectrum. The spectra were externally referenced to LiCl (aq) at 0.00 ppm.

All spectra were recorded and processed using Bruker Topspin 2.1, 3.6.2 and 3.6.4, fitted using DMfit software using the Chemical Shift Anisotropy (CSA) MAS/static model and analysed and plotted using home-written MATLAB scripts.^71^

### Charge–discharge measurements

Charge–discharge measurements were undertaken using a Biologic VMP3 cell tester with the cells held at 40 °C, cells were rested for at least 24 h prior to cycling. To assemble Li|Li_2_NH|LiFePO_4_ cells, Li metal was first applied to one side of a cold pressed pellet (10 mm diameter, 1.2 mm thickness, excess capacity = 38.2 mA h) before being clamped on a hot plate and held at 60 °C for 1 hour followed by 180 °C for 2 hours. Once cooled, the pellet would be placed in a CR2032 cell cap and 10 μL of 1M LiPF_6_ in EC : DMC with 2 wt% vinyl chloride (1 : 1, Sigma Aldrich) was added dropwise to the surface of the pellet. Commercially purchased LiFePO_4_ electrode sheets (Pi-KEM) with an active loading of 7.44 mg cm^−2^ (cut into 12 mm disks) were placed onto the stack with a steel spacer (0.5 mm thickness) and spring (0.25 mm thickness) followed by a CR2032 cell cap with an attached O-ring. The cell was then sealed using a Hohsen Corp coin cell crimper.

For Li|Li_2_NH|TiS_2_ cells, a cathode slurry was prepared by mixing 80 wt% TiS_2_ (99.9%, Sigma Aldrich, ball milled at 200 pm for 1 hour) as an active material, 8 wt% polyvinylidene fluoride (PVDF 5130, Solvay) as a binder and 12 wt% carbon black (TimCal, C65) as a conducting additive with *N*-methyl-2-pyrrolidone (NMP) as the solvent in a THINKY mixer at 2000 rpm for 15 minutes. The slurry was coated uniformly on aluminium foil using the doctor blade coating technique and dried in a vacuum oven at 120 °C for 24 h. The electrodes were cut into 12 mm disks for further use. The active material loading of the cathode was maintained at 3 mg cm^−2^. A cold-pressed Li_2_NH pellet of 10 mm diameter and 1.2 mm thickness was used as the SSE. Li metal was used as an anode and was melded on one side of the pellet as described above (37.9 mA h excess capacity). 10 μL of 1 M LiPF_6_ in EC/EMC (1 : 1) liquid electrolyte was again added while assembling the coin cells. The cells were discharged and charged at a current density of 5 mA g^−1^, cycling between 1.5 V and 3.2 V.

## Author contributions

JWM and JPL conceived the study and analysed the data. JPL conducted the experiments. PAA, PRS and EMK assisted with electrochemical data interpretation and experiment design. TVB collected and analysed the TiS_2_ cell data. TI and CPG designed experiments and analysed data from the solid-state NMR spectroscopy. SD assisted with collection of synchrotron X-ray diffraction data. MPS and BD assisted with the electrochemical experiments and associated analysis. JWM and JPL prepared the manuscript with input from all authors.

## Data availability

ESI† is available for this paper. Raw data supporting the figures presented in the paper can be accessed through the University of Birmingham e-data repository (https://doi.org/10.25500/edata.bham.00001212). Correspondence and requests for materials should be addressed to Dr Joshua Makepeace (j.w.makepeace@bham.ac.uk).

## Conflicts of interest

The authors declare no competing interests.

## Supplementary Material

EB-001-D5EB00058K-s001
