# Supplementary material for: Probing the electrochemical behaviour of lithium imide as an electrolyte for solid-state batteries
Source: EES Batter. 2025 Apr 2;1(3):527–40. doi: 10.1039/d5eb00058k (PMC12001454; doi:10.1039/d5eb00058k)
Supplement: EB-001-D5EB00058K-s001 [file EB-001-D5EB00058K-s001.pdf]

Supplementary Information (SI) for EES Batteries.
This journal is © The Royal Society of Chemistry 2025

Supplementary Information:

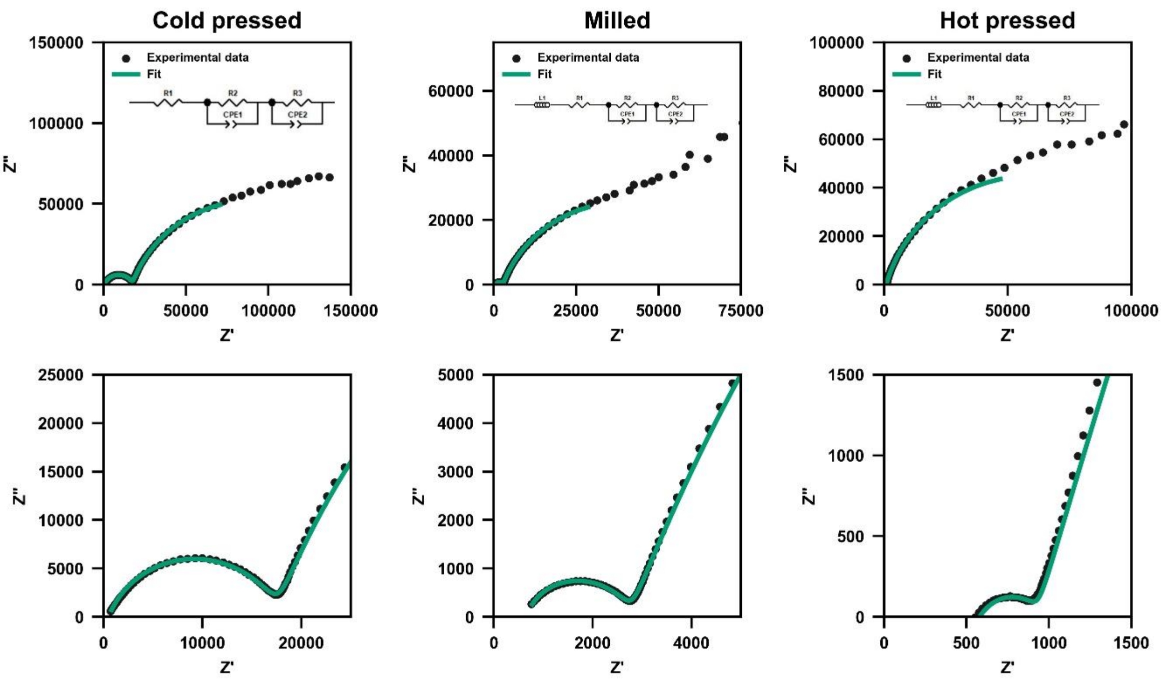

Supplementary Figure 1. Full (top) and zoomed (bottom) Nyquist plots of EIS data for Au|Li_2NH|Au cells with a) cold pressed Li_2NH b) milled Li_2NH and c) hot pressed Li_2NH, with equivalent circuit models indicated and the resultant fit to the data shown. R1 is a series resistance, with R2|CPE 1 representing the bulk+grain boundary conduction in lithium imide and R3|CPE representing the lithium imide – gold interface, characterised by a blocking spike. A bend in the spike is observed which is attributed to Li-Au alloying as observed when using sputtered Au in other studies.[70]

Supplementary Table 1. Results of the fitting of the EIS data for the Au|Li_2NH|Au cells displayed in Supplementary Figure 1.

	**L1 (H)**	**R1 (Ω)**	**R2 (Ω)**	**CPE1 (F s^{P-1})**		**R3 (Ω)**	**CPE2 (F s^{P-1})**	
				**T**	**P**		**T**	**P**
**Cold Pressed**	N/A	517 (13)	17040 (90)	1.03 (5)E-9	0.770 (3)	143780 (4899)	1.37(7)E-7	0.794 (6)
**Milled**	2.6 (1) E-6	415 (17)	2359 (22)	2.2 (2)E-9	0.735 (6)	72242 (874)	1.95(3)E-7	0.779 (2)
**Hot Pressed**	3.4 (1)E-6	360 (27)	570 (29)	4 (1) E-9	0.741 (2)	117490 (1409)	1.28(1)E-7	0.830 (1)

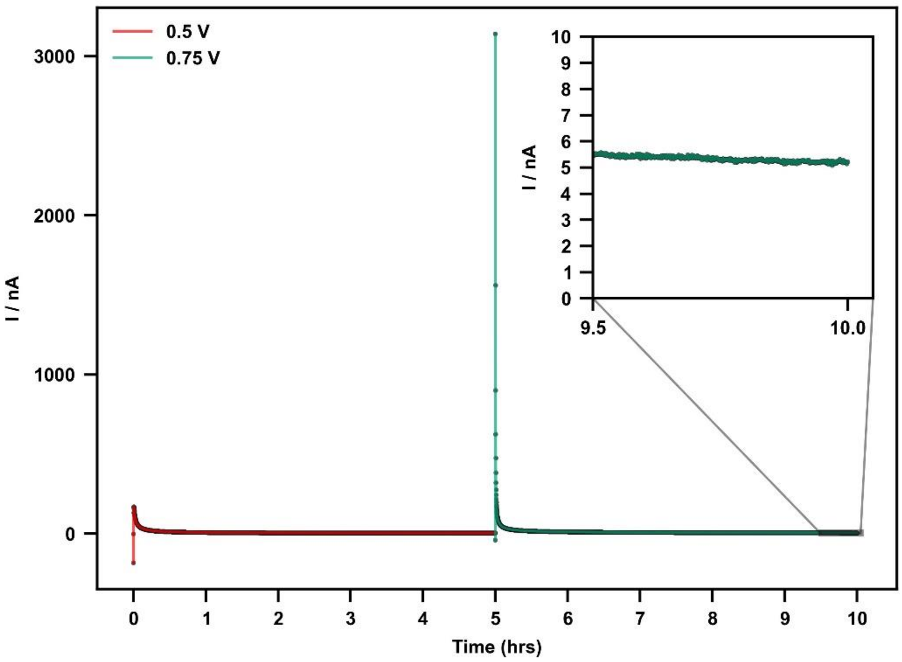

Supplementary Figure 2. DC polarisation data at 0.5 V and 0.75 V for an Au|Li_2NH|Au cell. An inset axis is displayed of the data in the last half hour of relaxation time under 0.75 V. The average current value over this period was calculated to be 5.3 nA. Pellet dimensions: radius (0.5 cm), width (0.132 cm).

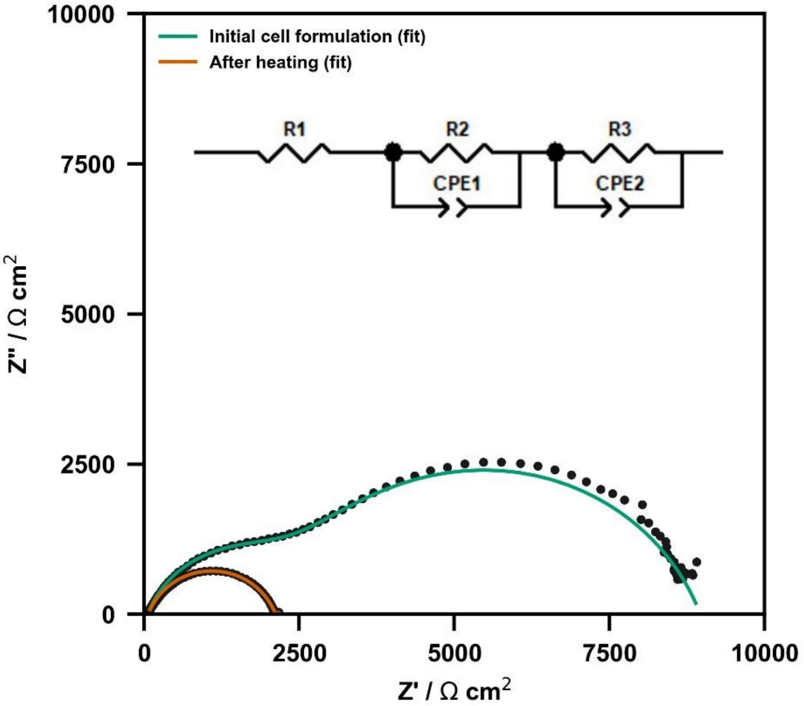

Supplementary Figure 3. Nyquist impedance spectra of a Li|Li_2NH|Li cell before and after heat treatment with equivalent circuit model indicated. Data is normalised using the surface area of the pellet to calculate the area specific resistance of the interface.

Supplementary Table 2. Results of the fitting of the EIS data for the Li|Li_2NH|Li cell displayed in Supplementary Figure 3.

	**R1 (Ω)**	**R2 (Ω)**	**CPE1 ($F\ s^{P-1}$)**		**R3 (Ω)**	**CPE2 ($F\ s^{P-1}$)**	
			**T**	**P**		**T**	**P**
**Initial cell formulation**	61	2288	6.8 E-9	0.790	6616	6.17 E-8	0.77
**After heating**	66	2047	7.9 E-9	0.779	94	7 E-4	0.56

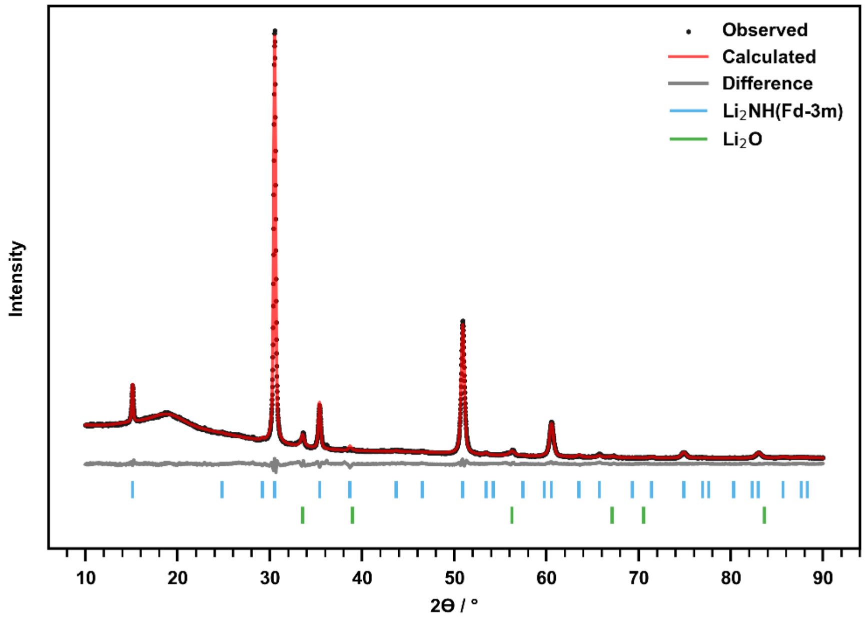

Supplementary Figure 4. X-ray diffraction pattern of lithium imide extracted from a Li|Li_2NH|Li cell after heat treatment to stabilise the lithium metal – lithium imide interface. The results of Rietveld analysis of the pattern are indicated.

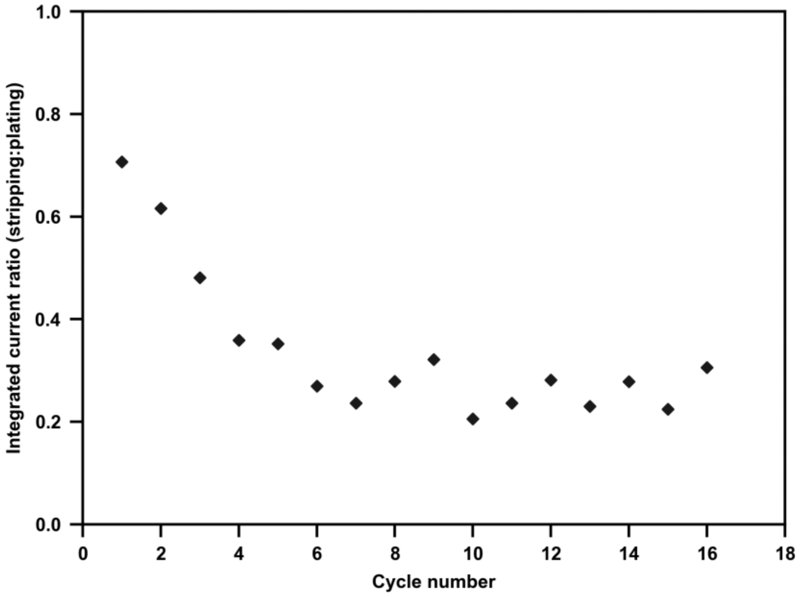

Supplementary Figure 5. The ratio of stripping current to plating current versus CV cycle number for a Li|Li_2NH|Steel coin cell.

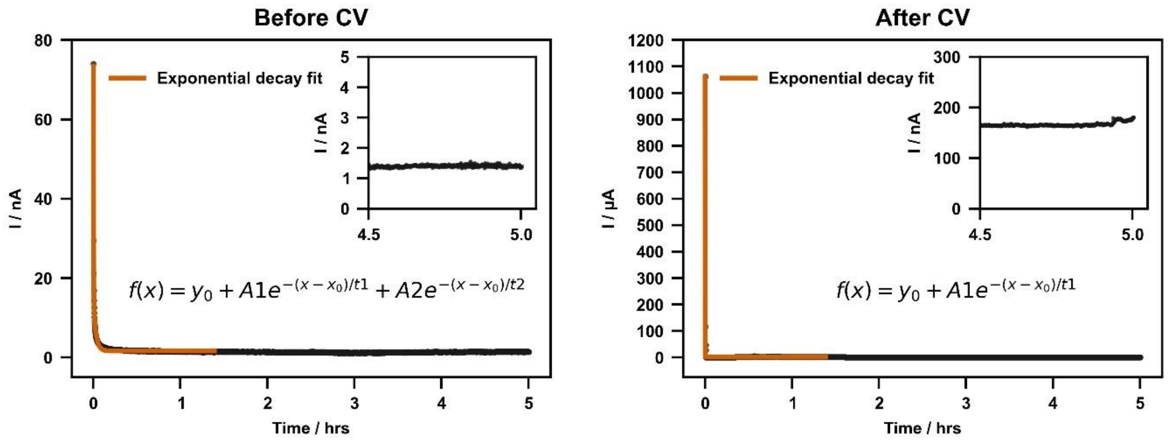

Supplementary Figure 6. DC polarisation experiment data (0.5 V) for a Li|Li_2NH|Steel coin cell before (left) and after (right) a CV experiment (16 cycles). Fits of exponential decay functions to both data sets are displayed in red with the equation used displayed in the centre. An inset axis is displayed of the data in the last half hour of relaxation time. The average current value over this period was determined to be 1.4 nA (Before CV) and 166 nA (After CV). Pellet dimensions: radius (0.5 cm), width (0.127 cm)

Supplementary Table 3. Results of the fitting of exponential functions to DC polarisation experiments before and after CV as displayed in Supplementary Figure 5. It should be noted that these numbers are used for the current measured in mA.

	y_0 (mA)	A1	x_0	t1 (s)	A2	t2 (s)
**Before CV**	1.64 (1) E-6	8.64	-38.26	4.48 (6)	1.93 E-7	105 (2)
**After CV**	0.00132 (5)	666.86	-14.67	2.27 (1)	N/A	N/A

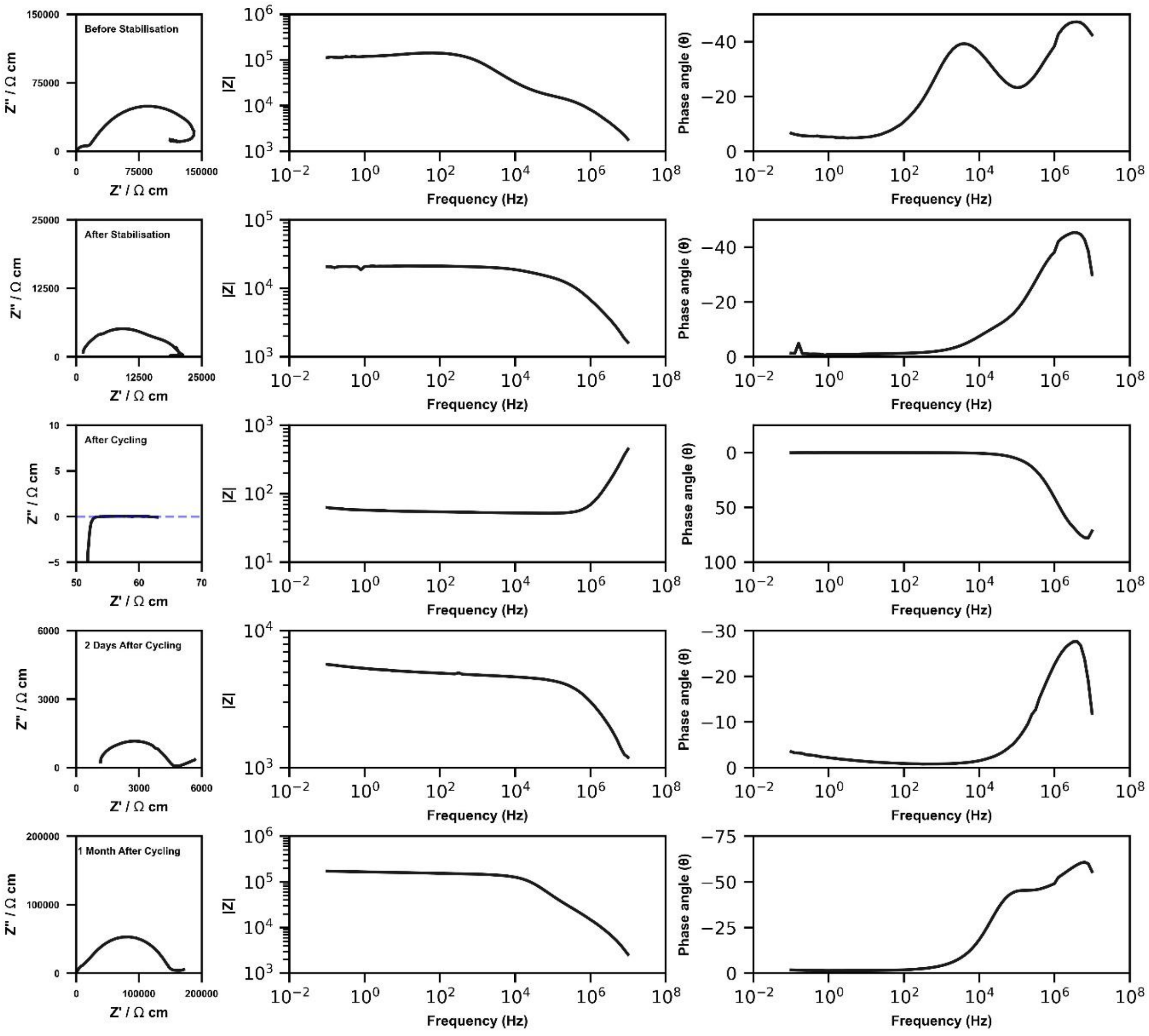

Supplementary Figure 7. Nyquist (left) and Bode (middle) and Phase angle (right) plots of EIS experiments on a Li|Li_2NH|Li coin cell at various stages through a Li stripping and plating experiment.

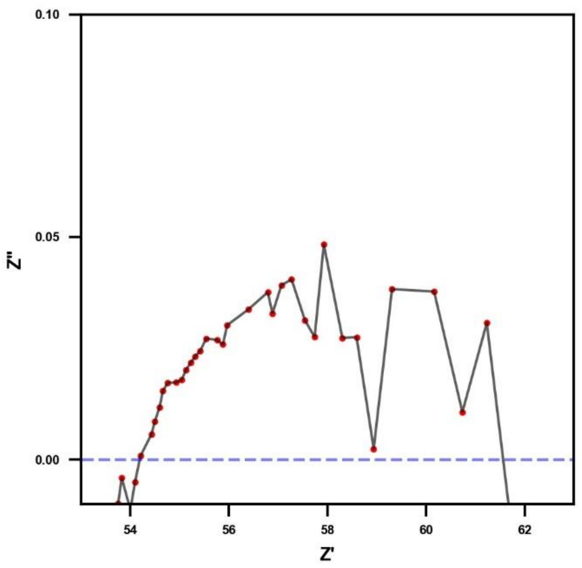

Supplementary Figure 8. Zoomed-in Nyquist plot of EIS data on a Li|Li_2NH|Li cell immediately post Li stripping and plating experiment.

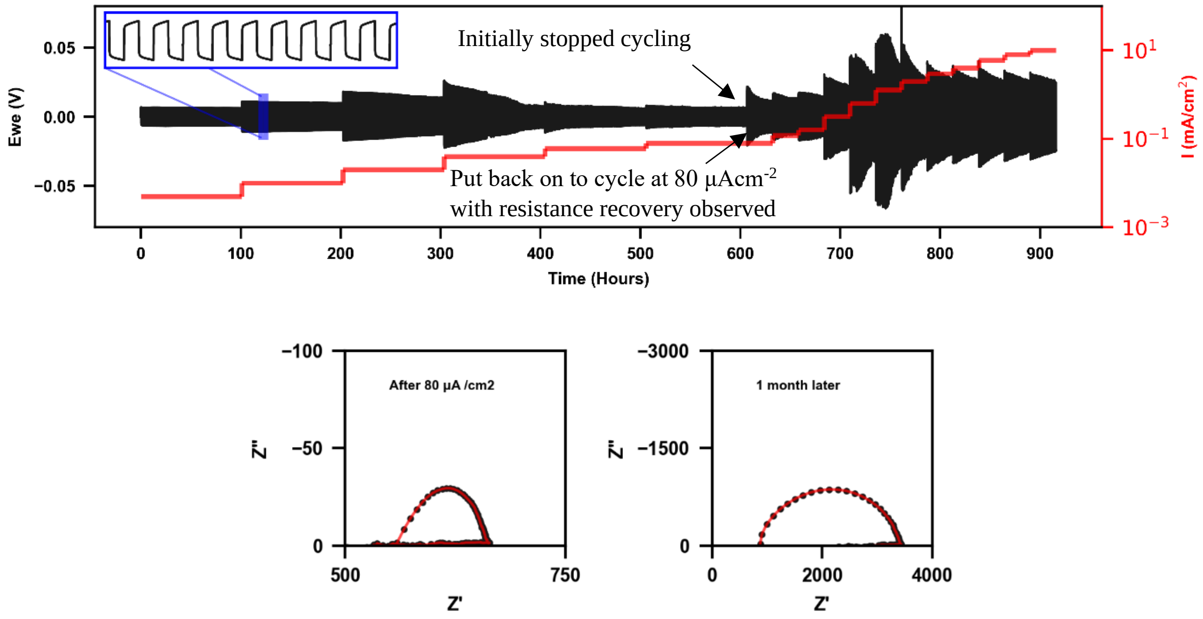

Supplementary Figure 9. Repeat experimental data of Li stripping and plating for a Li|Li_2NH|Li coin cell with EIS data showing resistance recovery. The positive and negative currents are not displayed so as to not obscure the voltage data, a line indicating the set current density is instead used alternating between positive and negative currents in 15-minute intervals.

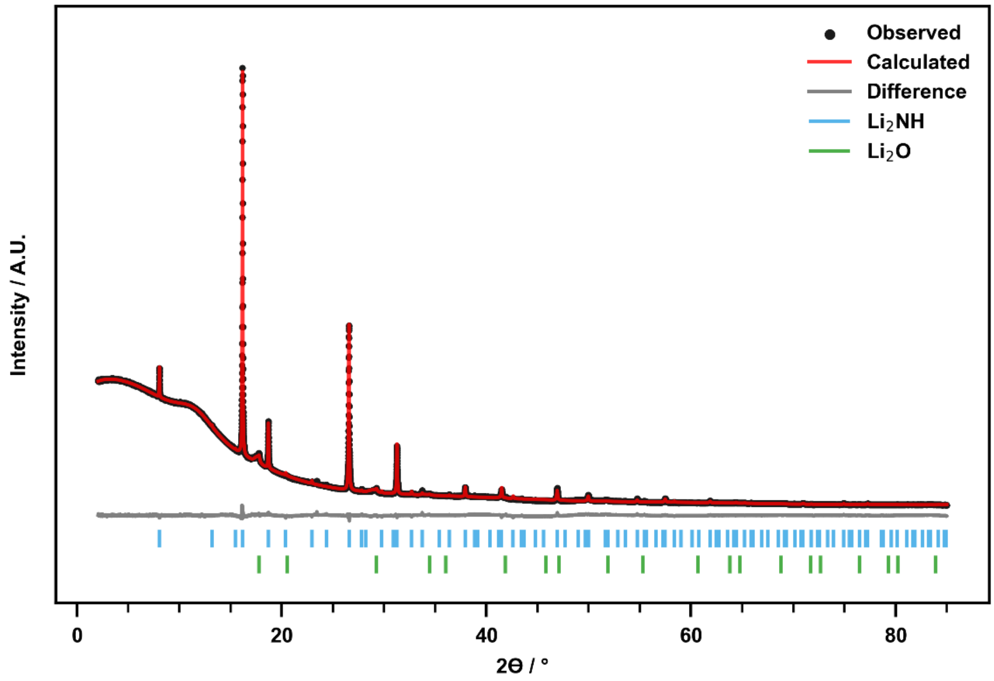

Supplementary Figure 10. XRD pattern of lithium imide taken from a Li|Li_2NH|Li coin cell after the Li stripping and plating experiment. Results of Rietveld analysis of the pattern are indicated.

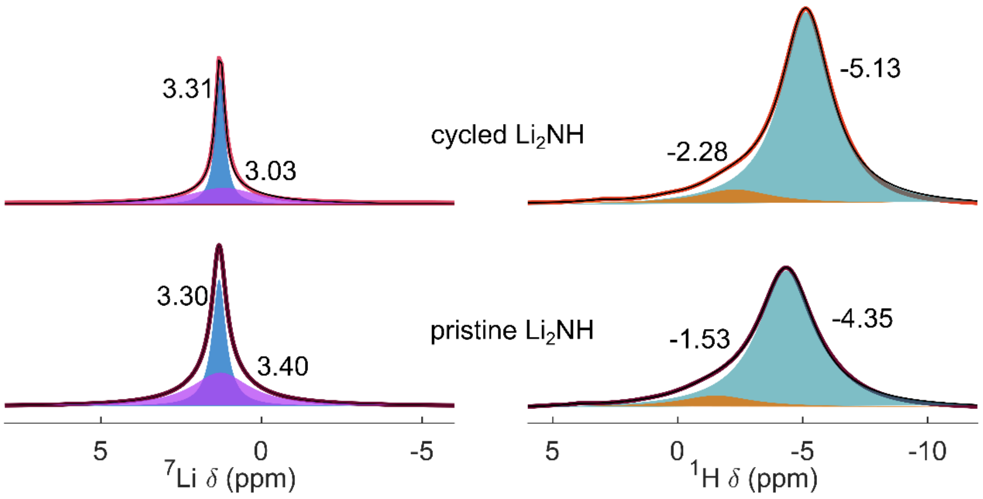

Supplementary Figure 11. ^{7}Li and ^{1}H solid state MAS NMR spectra of lithium imide (bottom) before and (top) after use in a Li|Li_2NH|Li Li stripping and plating experiment. The spectra are normalised by number of residuals and by the mass of the samples in the rotors.

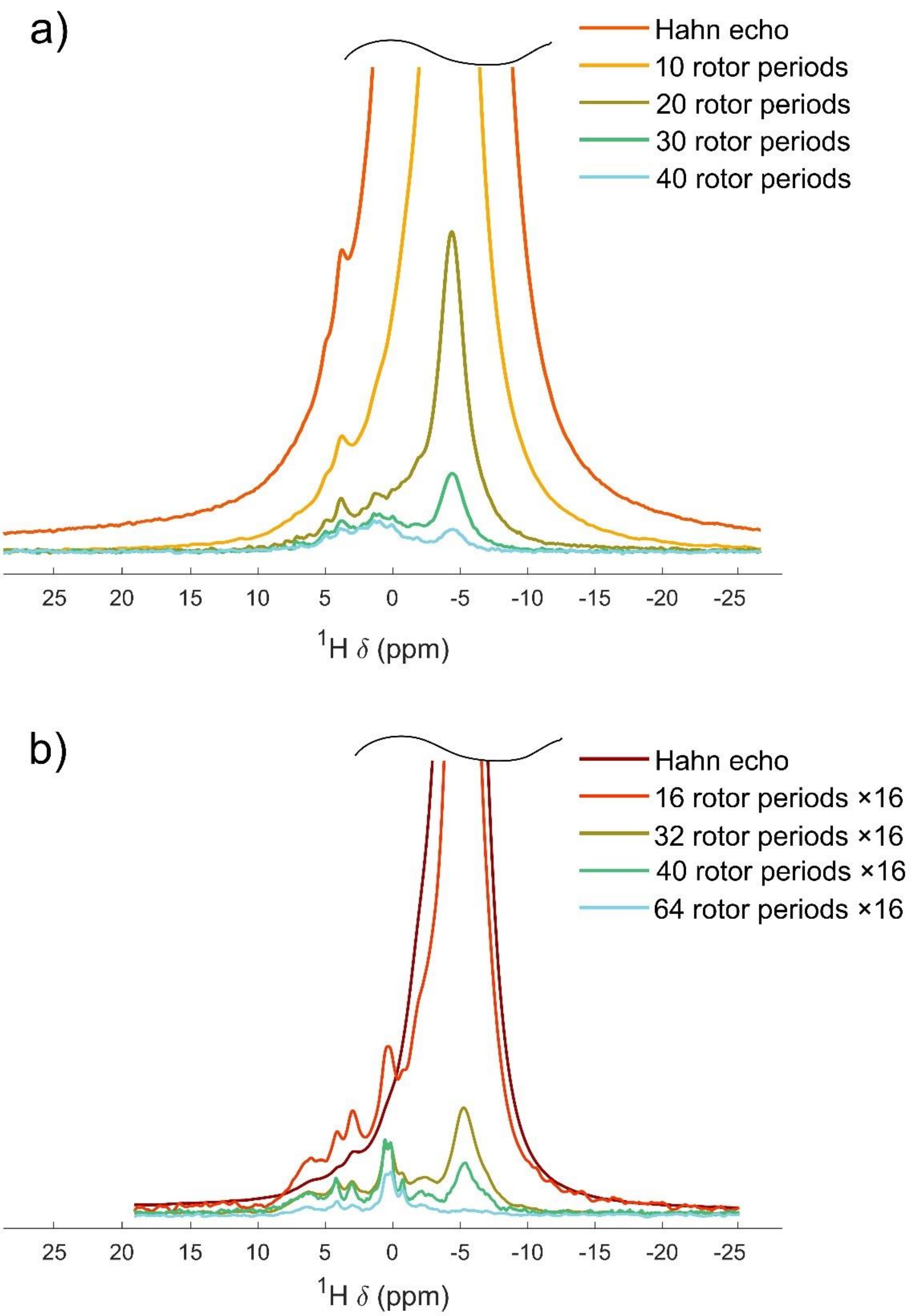

Supplementary Figure 12. ^{1}H solid state MAS NMR spectra of lithium imide (a) before and (b) after use in a Li|Li_2NH|Li Li stripping and plating experiment. The spectra were acquired by increasing the echo delay by integer numbers of rotor periods as labelled.

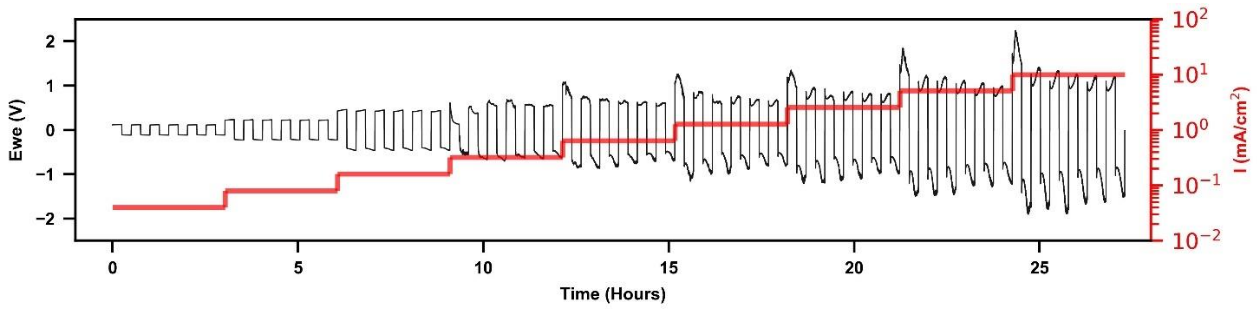

Supplementary Figure 13. Electrochemical data for the Li|Li_2NH|Li coin cell Li stripping and plating experiment on the I11 beamline. The positive and negative currents are not displayed so as to not obscure the voltage data, a line indicating the set current density is instead used alternating between positive and negative currents in 15-minute intervals.

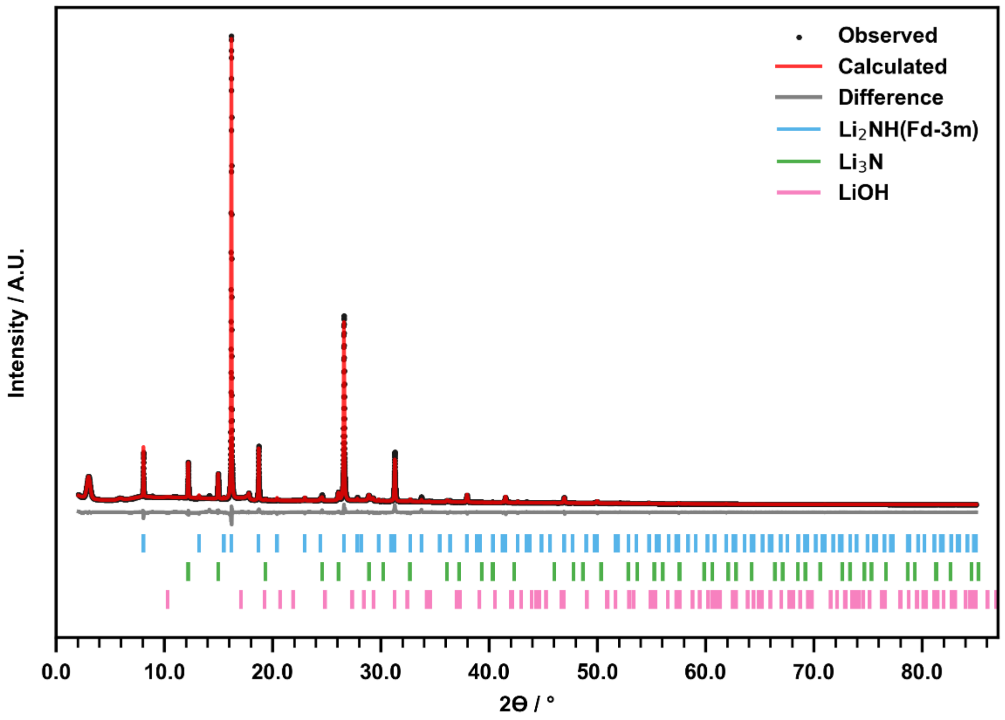

Supplementary Figure 14. Example X-ray diffraction data corresponding to the contour plot displayed in Figure 4a.

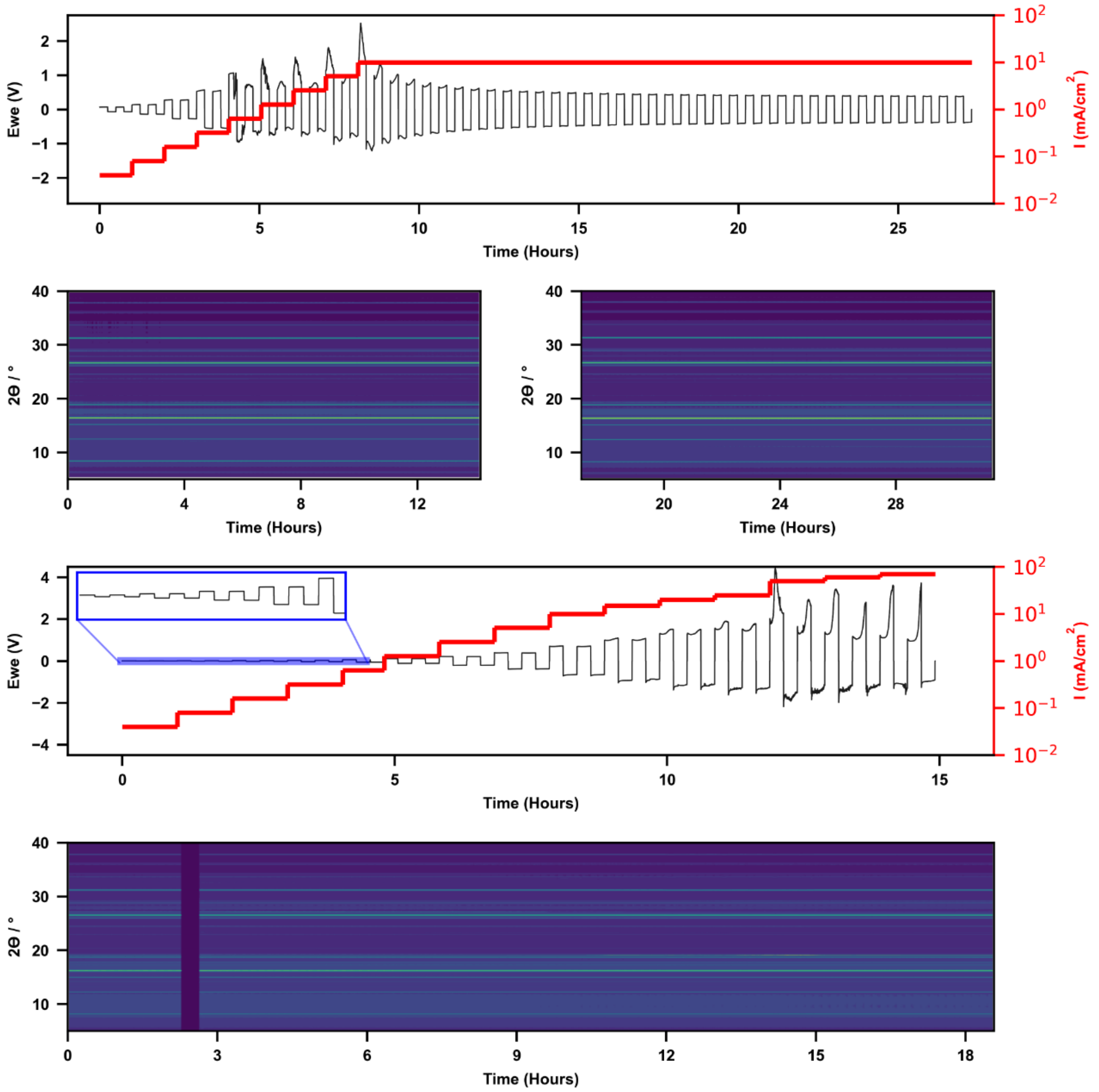

Supplementary Figure 15. Electrochemical data (top) and contour plot of the X-ray diffraction data (bottom) for the second Li|Li_2NH|Li coin cell Li stripping and plating experiment on the I11 beamline. The positive and negative currents are not displayed in the stripping and plating experiments so as to not obscure the voltage data, a line indicating the set current density is instead used alternating between positive and negative currents in 15-minute intervals.

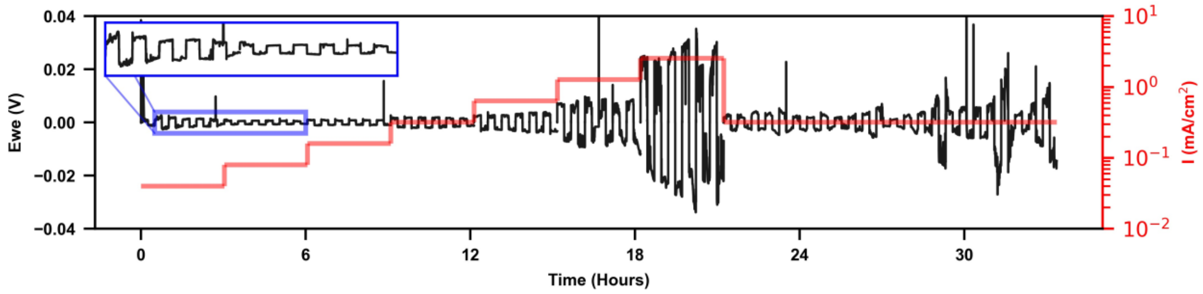

Supplementary Figure 16. Electrochemical data for the Li|Li_2NH|Li coin cell Li stripping and plating experiment during the *in situ* solid state NMR experiment. The positive and negative currents

are not displayed so as to not obscure the voltage data, a line indicating the set current density is instead used alternating between positive and negative currents in 15-minute intervals.

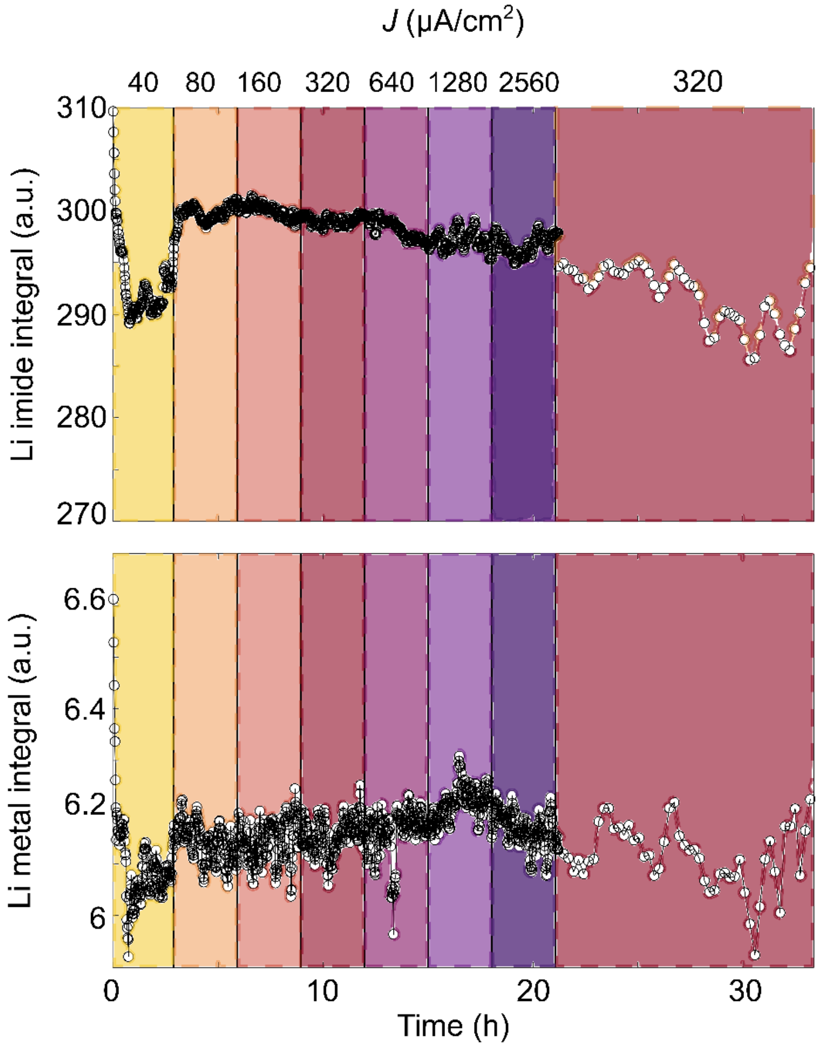

Supplementary Figure 17. Integrated solid state NMR peak intensities for lithium imide and lithium metal during the Li|Li_2NH|Li coin cell Li stripping and plating experiment.

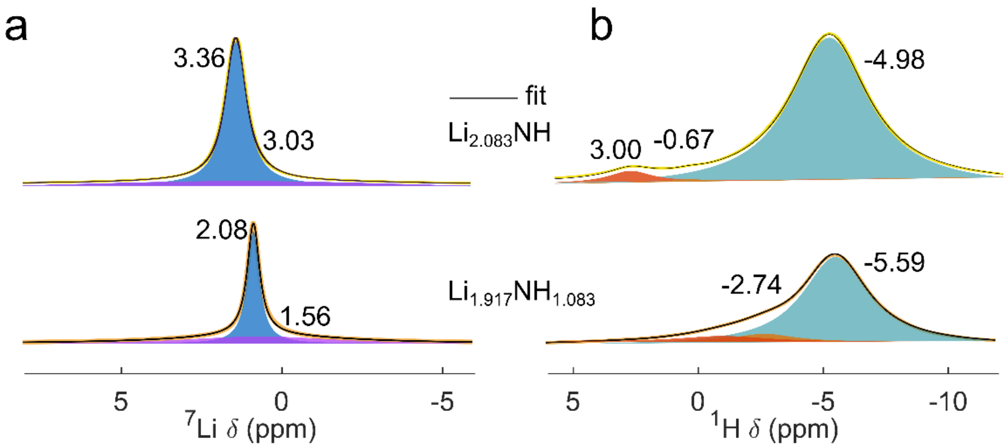

Supplementary Figure 18. ⁷Li and ¹H solid state MAS NMR spectra of a lithium imide-amide sample ($Li_{1.917}NH_{1.083}$, bottom) and a lithium imide-nitride-hydride sample ($Li_{2.083}NH$, top).

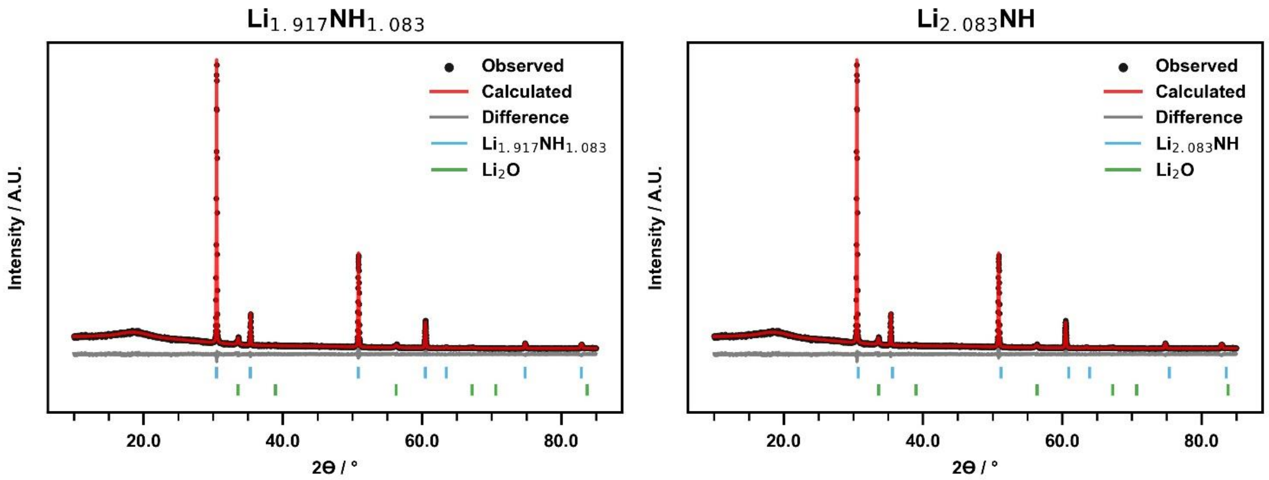

Supplementary Figure 19. Rietveld refinement of X-ray diffraction patterns of the lithium imide-amide ($Li_{1.917}NH_{1.083}$) and lithium imide-nitride-hydride samples ($Li_{2.083}NH$). Data collected on a Bruker D5000 instrument.

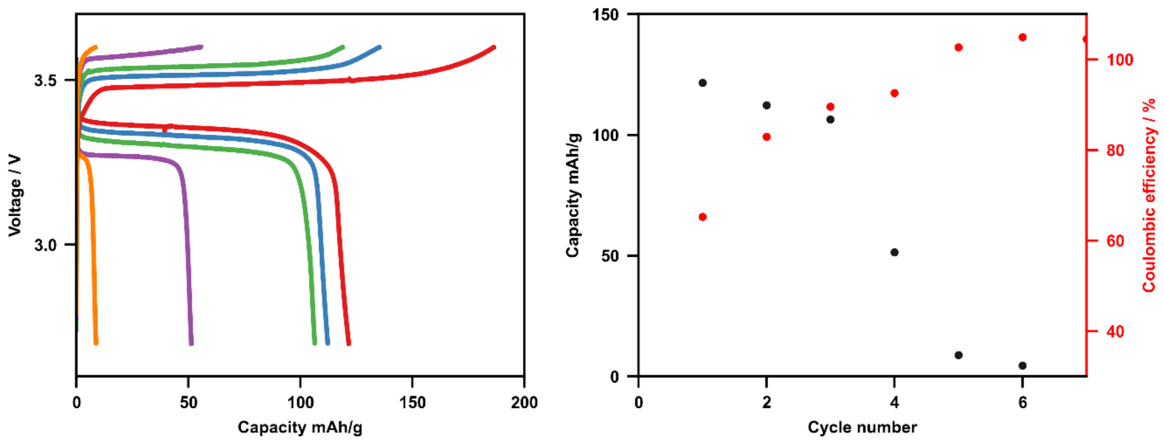

Supplementary Figure 20. Cell cycling data for a Li|Li_2NH|LFP full cell with no VC additive present in the liquid electrolyte.
